# Stability convergence in natural antibodies with ultra-long hypervariable loops

**DOI:** 10.1038/s42003-025-08036-5

**Published:** 2025-04-19

**Authors:** Marcel Passon, Matja Zalar, Thomas Nehls, Stefaan De Smedt, Frederik Lermyte, Ahmad Reza Mehdipour, Hristo L. Svilenov

**Affiliations:** 1 Faculty of Pharmaceutical Sciences, Ghent University, Ottergemsesteenweg 460, 9000 Ghent, Belgium; 2 Faculty of Chemistry and Chemical Engineering, University of Maribor, Maribor, Slovenia; 3 Clemens-Schöpf Institute of Organic Chemistry and Biochemistry, Department of Chemistry, Technical University of Darmstadt, Darmstadt, Germany; 4 Center for Molecular Modeling, Ghent University, Ghent, Belgium; 5 Biopharmaceutical Technology, TUM School of Life Sciences, Technical University of Munich, Emil-Erlenmeyer-Forum 5, 85354 Freising, Germany

**Keywords:** Antibody therapy, Recombinant protein therapy

## Abstract

Antibodies bind to antigens with hypervariable loops called complementarity-determining regions (CDRs). In contrast to conventional antibodies, a subset of bovine antibodies has an ultra-long CDR (ulCDR) composed of up to 70 residues folded as a stalk and knob. The fundamental principles of how these antibodies maintain their structure and stability remain enigmatic. Here, we investigated how different natural ulCDRs affect antibody structure, stability and function. To this end, we swapped diverse ulCDRs onto the same antibody scaffold. All ulCDR-swap variants exhibit nearly identical secondary structure fingerprints and remarkably similar thermal stability. In addition, specificity and high-affinity binding to the antigens are maintained. Hydrogen-deuterium exchange and molecular dynamics simulations suggest small differences between the variants arising from changed interactions between different stalks and the underlying scaffold. Overall, we reveal principles of grafting natural ulCDRs onto a common Fab scaffold, which have implications for antibody design for biomedical applications.

## Abstract (web summary)

Diverse ultra-long CDRs can be swapped on the same antibody scaffold with minimal impact on scaffold structure and stability.

## Introduction

Antibodies are essential tools of the adaptive immune system. Conventional antibodies consist of two heavy (HC) and two light chains (LC) assembled into a distinctive Y-shaped structure^1^. The HCs and LCs consist of constant and variable domains with a characteristic immunoglobulin fold. Antibody specificity is conferred by the variable domains that contain hypervariable loops called complementarity-determining regions (CDRs). There are three CDRs in the HC (CDR-H1, CDR-H2, CDR-H3) and three in the LC (CDR-L1, CDR-L2, CDR-L3). In most antibodies, several CDRs form the antigen-binding site.

Despite their hypervariability, canonical CDRs are limited in length. For example, human CDR-H3s typically consist of only 6–20 amino acids^2,3^. In striking contrast, some bovine antibodies have an ultra-long CDR-H3 (ulCDR) made of up to 70 amino acids^4–6^. The ulCDR is folded into a stalk and a knob mini-domain^7^. The knob exhibits immense sequence and structural diversity due to different disulfide bond patterns^7,8^. Remarkably, the bovine knobs contain all residues needed for binding to the antigen and can be produced as isolated peptides with a molecular mass of only 4–5 kDa^9–12^. The stalk serves as a bridge that connects the knob to the antibody framework^9,10,13^. The HCs of ulCDR-antibodies pair with a restricted set of LCs^14^. The conserved LCs in ulCDR-antibodies do not contribute to the antigen-binding site, but it was recently shown that some of these LCs exhibit very favorable physicochemical properties that contribute to the stability of the antibody^15,16^.

Interestingly, it is possible to remove the entire knob region from the ulCDR without negatively affecting antibody stability and secretion from mammalian cells^9^. In contrast, replacing the bovine stalk with glycine residues dramatically reduces the Fab melting temperature (*T*_m_) by ~12 °C and diminishes secretion by mammalian cells^9^. Therefore, it appears that the stalk is an essential structural element that enabled the evolution of a knob mini-domain coupled to an antibody framework. In addition, it is possible to graft a knob mini-domain onto human Fabs, and the binding affinity of the knob-graft is higher when the human scaffold has a stalk-like long CDR-H3, thus supporting the notion that knob mini-domains are a separate functional entity^9^.

While the research on the versatility of the knob domains, also called picobodies, is rapidly progressing^12,17–22^, little is known about how natural stalks govern ulCDR-antibody structure and stability. In particular, it is puzzling whether different natural ulCDRs are fully compatible with the same Fab scaffold.

Here, we sought to understand the impact of different natural ulCDRs on antibody structure and stability. To this end, we designed diverse ulCDR-swap mutants using the same bovine Fab scaffold. We discovered surprisingly similar secondary structure fingerprints and thermal stability in antibody variants with divergent natural ulCDRs grafted on the same Fab scaffold. Furthermore, antigen binding was preserved upon ulCDR-swapping with little to no affinity loss compared to the parent ulCDR-antibodies.

Overall, these findings suggest that natural ulCDRs can be exchanged on a common Fab scaffold to obtain antibodies with different antigen specificities but similar scaffold secondary structure and thermal stability.

## Results

### Fab variants with different ulCDRs exhibit convergence of their thermal stabilities

**Fig. 1:**
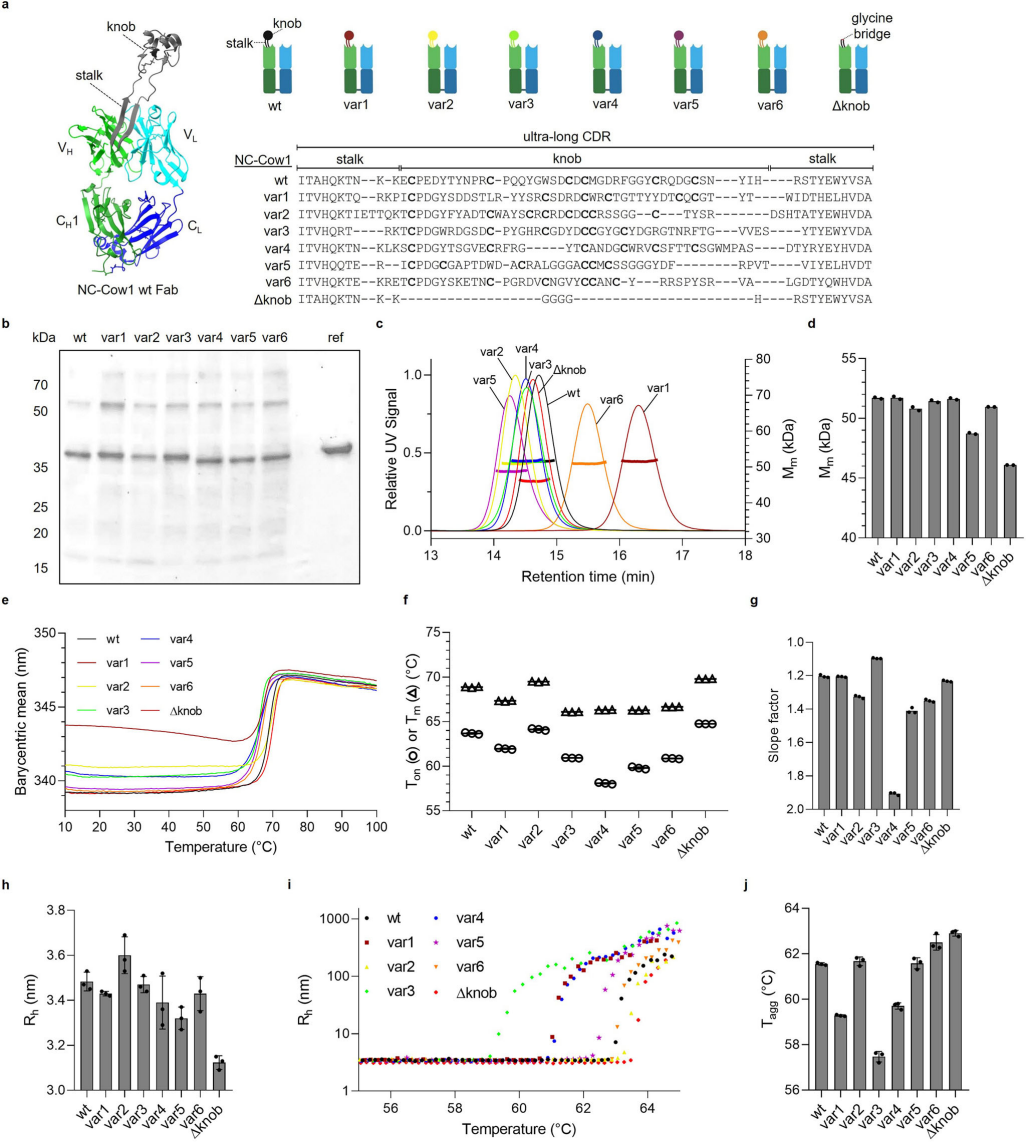
Design, secretion, homogeneity, and stability of Fabs with swapped ultra-long CDR loops. **a** Structure of a bovine Fab NC-Cow1 (PDB:6OO0) with an ultra-long CDR-H3 (gray color). Schematic figure and ulCDR sequence alignment of the Fab variants in this work. **b** Non-reducing SDS-PAGE of cell supernatants with secreted Fab fragments and a purified NC-Cow1 wt reference. **c** Chromatograms and molecular mass in SEC-MALS. **d** Mean values (*n* = 2) of molecular masses calculated from MALS. **e** Exemplary melting curves in SUPR-DSF. **f** Apparent onset temperatures of unfolding *T*_on_ and melting temperatures *T*_m_ obtained from SUPR-DSF measurements. Mean values (*n* = 3). **g** Slope factors of fitted Boltzmann functions obtained from SUPR-DSF melting curves. A smaller slope factor indicates a steeper transition (more cooperative unfolding). Mean values (*n* = 3; SD shown in Table 1). **h** Apparent hydrodynamic radii *R*_h_ from DLS. Mean values (*n* = 3) with SD. **i** Apparent hydrodynamic radii *R*_h_ during heating ramp with DLS. **j** Aggregation onset temperatures *T*_agg_ obtained from heat-ramped DLS. Mean values (*n* = 3) with SD.

We were interested in how different ultra-long CDRs affect antibody stability. To this end, we selected seven natural ultra-long CDRs with low sequence homology and diverse properties (Supplementary Table 1; Supplementary Fig. 1). Next, we exchanged the ulCDR in a model bovine Fab (NC-Cow1 wt) with the other six ulCDRs (Fig. 1a). This resulted in Fab variants where approximately 10% of the protein sequence was different. In addition, we included a truncated variant (Δknob) where the knob residues in NC-Cow1 wt were replaced with four glycine residues^9^. All six ulCDR-swap Fab variants and the wt were secreted well by mammalian cells (Fig. 1b). We then purified the Fabs to homogeneity for further characterization. SEC-MALS and SDS-PAGE revealed that the proteins are monodisperse with the expected molecular masses (Fig. 1c, d; Table 1; Supplementary Fig. 2). When comparing the variants with hydrophobic interaction chromatography (HIC), we found that exchanging the ulCDRs leads to shifts in HIC retention times (Supplementary Fig. 3). The differences in peak shapes and retention times underline the impact of the ulCDR on the hydrophobicity of the Fab. The variant without a knob showed the shortest retention time in HIC (Supplementary Fig. 3). To compare the stability of the Fabs, we first assayed the variants with differential scanning fluorimetry in microwell plates (DSF). The analysis revealed steep two-state unfolding transitions within a narrow temperature range, yielding apparent melting temperatures (*T*_M_s) between 66 and 70 °C (Fig. 1e, f; Table 1). Further analysis of the onset temperatures of unfolding (*T*_on_s) and the slopes of the melting curves showed that var4 exhibits the least cooperative unfolding compared to the other variants (Fig. 1f, g). To gain further insights into anticipated differences between the variants, we used dynamic light scattering (DLS). The apparent hydrodynamic radius (*R*_h_) of all variants is close to the theoretical value of ~3.3 nm calculated^23^ from the 3D structure of NC-Cow1 wt (Fig. 1h). Var2, with the longest stalk, has the highest *R*_h_, while the Δknob variant has the smallest *R*_h_ (Fig. 1h; Table 1). Next, we performed temperature-ramped DLS measurements to investigate differences in the aggregation of the variants. All proteins have similar aggregation profiles during heating and aggregation onset temperatures (*T*_agg_s) spreading over a narrow temperature interval, 57–63 °C, with Δknob having the highest *T*_agg_ (Fig. 1j; Table 1). Therefore, these results indicate that the thermal stability of the tested Fab variants is very similar despite the very different ulCDRs.

**Table 1:** Overview of physicochemical descriptors of NC-Cow1 Fab and ulCDR mutants

Construct	*M*_m,theoretical_ (kDa)	*M*_m,MALS_ (kDa)	*T*_on_ (°C)	*T*_m_ (°C)	*R*_h,DLS_ (nm)	*T*_agg_ (°C)	*K*_D_ (nM)
wt	51.8	51.7 ± 0.1	63.7 ± 0.1	68.9 ± 0.1	3.5 ± 0.0	61.6 ± 0.1	2.6
var1	51.9	51.7 ± 0.2	62.0 ± 0.1	67.3 ± 0.1	3.4 ± 0.0	59.3 ± 0.0	125
var2	51.5	50.8 ± 0.2	64.1 ± 0.1	69.5 ± 0.0	3.6 ± 0.1	61.7 ± 0.2	118
var3	51.5	51.4 ± 0.1	60.9 ± 0.0	66.1 ± 0.0	3.5 ± 0.0	57.5 ± 0.2	14
var4	51.6	51.6 ± 0.1	58.1 ± 0.1	66.3 ± 0.0	3.4 ± 0.1	59.7 ± 0.1	43
var5	50.4	48.7 ± 0.1	59.8 ± 0.1	66.3 ± 0.0	3.3 ± 0.1	61.6 ± 0.2	31
var6	51.3	51.0 ± 0.0	60.9 ± 0.0	66.7 ± 0.0	3.4 ± 0.1	62.5 ± 0.3	77
Δknob	47.3	46.1 ± 0.0	64.8 ± 0.0	69.8 ± 0.0	3.1 ± 0.0	62.9 ± 0.1	n.b.

Mean values with standard deviation are shown. *n.b*. no binding.

To further investigate the importance of the Fab scaffold in antibodies with ulCDRs, we also produced the parent Fabs that harbor the ulCDRs of var1 (NC-Cow2^13^, here labeled var1PA), var3 (60E11^24^, here labeled var3PA) and var4 (60H05^24^, here labeled var4PA) (Supplementary Fig. 4). We then analyzed the thermal unfolding and aggregation profiles of the parent Fabs with DSF and DLS (Supplementary Fig. 4). The parent Fabs exhibit 4–8 °C higher T_M_ and 5–10 °C higher *T*_agg_ values compared to the corresponding ulCDR variants (Supplementary Fig. 4; Supplementary Table 2). This indicates that the Fab scaffold itself has a larger impact on the overall Fab stability than the swapping of the different natural ulCDRs on a common Fab.

Collectively, these results reveal that swapping entire natural bovine ulCDRs on the same Fab scaffold has only a minor impact on the thermal stability of the Fab.

### Swapping of ulCDRs results in Fab fragments with preserved antigen binding

**Fig. 2:**
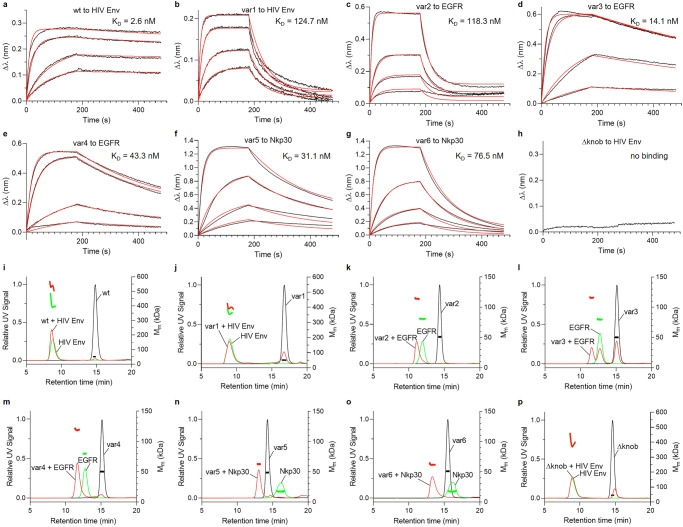
Target binding of NC-Cow1 Fab wt and variants with swapped ulCDR. **a**–**h** Kinetic measurements of Fab fragments against their respective antigens (HIV Env trimer for wt and var1 as well as Δknob, human EGFR for var2, var3 and var4, human Nkp30 for var5 and var6). The antigen binding was measured at different Fab concentrations (50, 100, 500, 1000 nM for wt, var 3 and var4. 100, 200, 500, 1000 nM for var1. 1000 nM for Δknob. 25, 50, 100, 500 nM for var2, var5, and var6.) and fitting functions (in red color) were calculated with the BLItz software. **i**–**p** Molecular mass and eluting peaks in SEC-MALS for Fab variants (black), their corresponding antigens (green), and Fab/antigen mixtures (red). Samples with Fab and HIV Env trimer were mixed in a 3:1 ratio, while the samples with Fab and EGFR or Nkp30 were mixed in a 1:1 ratio.

The correct folding of the knob in the ulCDRs is essential for target binding^9,18^. We, therefore, asked whether our ulCDR-swap variants bind to their antigens. To this end, we used biolayer interferometry (BLI) to probe the interaction between the Fabs and their corresponding antigens (Fig. 2a–h). The antigens are a soluble HIV envelope trimer for wt and var1^13^, human EGFR for var2, var3, and var4^24^, and human Nkp30 for var5 and var6^25^. All six Fabs with a swapped ulCDR retain binding to their target with nanomolar affinity (Fig. 2a–g, Table 1). The highest binding affinity was measured for the wt/HIV Env pair (*K*_D_ = 2.6 nM), while the lowest affinity was observed for the var1/HIV Env interaction (*K*_D_ = 125 nM). In contrast, the Δknob variant does not bind to the antigens (Fig. 2h, Supplementary Fig. 5a, b). To test if ulCDR-swapping has a negative impact on binding affinity, we also measured antigen binding of the parent Fabs of var1, var3, and var4. The binding affinity was only slightly lowered for var1 (125 nM vs 81 nM), whereas it remained unaffected for var3 (14 nM vs 15 nM) and var4 (43 nM vs 41 nM) compared to their respective parent Fabs (Supplementary Fig. 6a–c; Supplementary Table 2).

To gain insights into the stoichiometry of the Fab-antigen complexes, we used SEC-MALS (Fig. 2i–p). When incubating the wt with HIV Env trimer (3:1 ratio), we observed complexes with a mass corresponding to one HIV Env trimer and two or three Fab fragments (Fig. 2i), in agreement with previous findings that up to three NC-Cow1 Fabs can simultaneously bind to the HIV Env trimer^9,13^. In contrast, the complex of var1 incubated with HIV Env trimer (3:1 ratio) could be attributed to 1:1 stoichiometry (Fig. 2j). To verify that this difference in binding stoichiometry compared to wt is not a result of ulCDR-swapping, the same experiment was conducted with the parent Fab of var1 (var1PA), and we observed a similar complex formation corresponding to approximately 1:1 stoichiometry (Supplementary Fig. 6d). Furthermore, we measured complexes with masses corresponding to equimolar interactions for variants 2–4 with recombinant monomeric EGFR and for variants 5 and 6 with recombinant monomeric Nkp30. Interestingly, despite a high binding affinity measured in BLI (*K*_D_ = 14 nM), var3 contained a significant fraction of Fab that did not bind to its antigen when the components were mixed in an equimolar ratio (Fig. 2l). To explore if the unactive Fab fraction was formed due to the ulCDR-swapping, we produced the parent Fab of var3 (var3PA) and found that also a major fraction of the parent Fab did not form a complex with EGFR (Supplementary Fig. 6e). To test if the unbound fractions of var3 and var3PA have a nonfunctional ulCDR, we collected these fractions and found that they do not bind to EGFR in BLI (Supplementary Fig. 7a–d). Interestingly, the thermal stability of the inactive var3 and var3PA fractions is comparable to the purified antibodies containing functional Fabs (Supplementary Fig. 7e, f).

Lastly, Δknob does not form complexes with the antigens (Fig. 2p; Supplementary Fig. 5c, d), indicating, as expected, that target binding is abolished when the knob is removed.

In conclusion, these results show that natural ulCDRs exhibit high-affinity target binding when swapped on a common antibody scaffold.

### Fab secondary structure fingerprints are preserved upon ulCDR-swapping

**Fig. 3:**
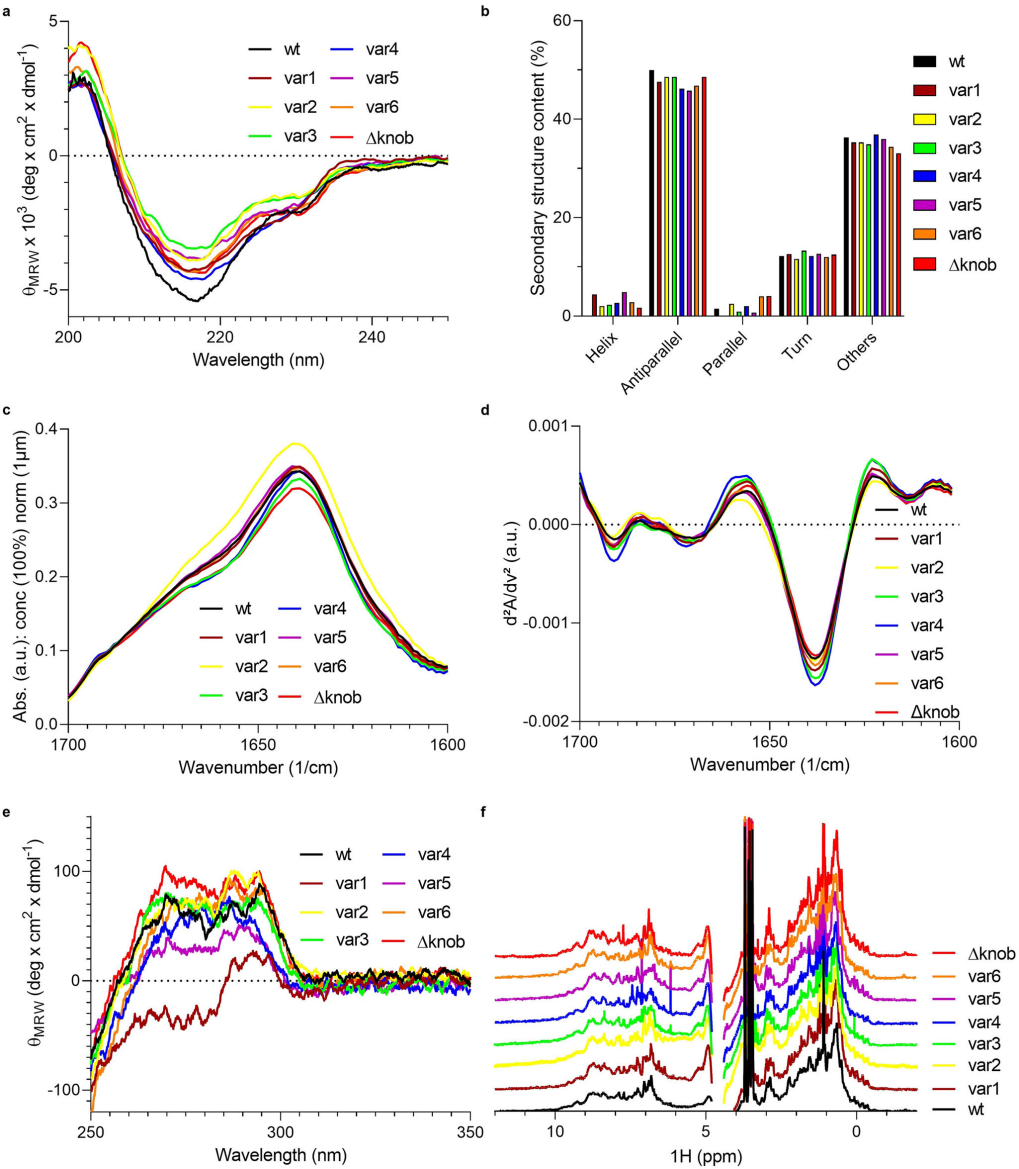
Structural comparison of NC-Cow1 Fab wt and ulCDR mutants. **a** Far-UV CD spectra. **b** Calculated secondary structure elements from Far-UV CD. The secondary structure elements were calculated using BestSel^39,57^. **c** Normalized absolute mid-wavelength IR spectra using MMS. **d** Second derivative spectra of MMS measurements shown in (**c**). **e** Near-UV CD spectra. **f** 1H NMR spectra of the different variants. Amide and aromatic range (6.5–10 ppm) and methyl-aliphatic range (−1 to 3.5) were used in the analysis, while the signals between 3.5 and 4.8 ppm were excluded due to the overlay of protein signals with water and glycerol signals.

We became interested in the structural differences between constructs after observing that the ulCDR-swap variants exhibit similar stability and preserved target binding. We used far-UV (FUV) circular dichroism (CD) to assay the secondary structure and observed nearly identical FUV-CD fingerprints with a negative peak at around 218 nm and a positive peak at around 200 nm, typical for beta-sheet proteins (Fig. 3a)^26^. We calculated the secondary structure elements from the FUV-CD data and obtained very similar results for the eight variants (Fig. 3b). In addition, we performed microfluidic modulation spectroscopy (MMS) to further investigate secondary structure (Fig. 3c). The second derivatives from the MMS spectra in the amide I region show high similarity between the samples (Fig. 3d), indicating that ulCDR-swapping has minor effects on the overall antibody structural fingerprint.

To assay the tertiary structure, we used near-UV CD spectroscopy and observed minor spectral differences likely due to variations in the aromatic residues and disulfide bonds in the ulCDRs with the biggest spectral differences exhibited by var1 and Δknob (Fig. 3e)^26^.

Finally, we performed proton nuclear magnetic resonance (1H NMR) and detected small variations in the aromatic and methyl regions, likely due to the different ulCDR regions (Fig. 3f). However, the overall 1H NMR structural fingerprints of the variants show a high correlation with each other (Supplementary Fig. 8).

Overall, these results indicate that ulCDR-swapping preserves the structural integrity of the Fab framework and that the overall structural fingerprint of the bovine Fab scaffold obtained with low-resolution methods is not affected by different natural ulCDRs.

### Structural consensus between the stalk and surrounding CDR loops is important for stability

**Fig. 4:**
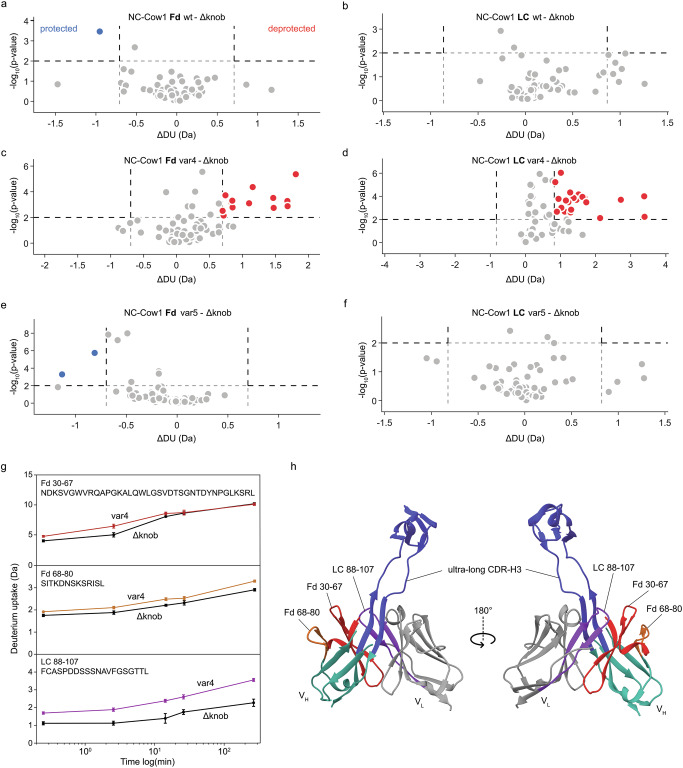
Deuterium uptake of NC-Cow1 Fab wt and variants with swapped ulCDR. **a**–**f** Volcano plots of differential deuterium uptake of peptides of wt, var4, and var5 in comparison with Δknob, shown for the Fd region and the LC. Peptides with significant protection compared to Δknob are marked in blue, and peptides with significant deprotection are marked in red. ∆knob was taken as the reference sample because it exhibits the highest stability. The thresholds for statistically significant differences were calculated for each specific dataset with Deuteros 2.0, as explained^58^. **g** Deuterium uptake plot over time for three peptides with a significant deprotection in var4 compared to Δknob. Mean values (*n* = 5) with standard deviation. **h** Visualization of the three peptides shown in (**g**) (same colors) on the crystal structure of NC-Cow1 wt (PDB:6OO0).

To unveil the mechanistic reasons for the subtle stability variations between our ulCDR-swapped variants, we sought to probe the protein structural dynamics. To this end, we used hydrogen-deuterium exchange coupled with mass spectrometry (HDX-MS) to determine the region-specific deuterium uptake in NC-Cow1 wt, Δknob, var4 and var5 (Fig. 4, Supplementary Fig. 9–16). The comparison between wt and Δknob enabled us to study the impact of the knob on the Fab, while var4 and var5 were selected because they exhibit slightly lower unfolding onset temperatures (Fig. 1f). Remarkably, when comparing Δknob with wt and var5, there were only minor differences in the deuterium uptake (Fig. 4a, b, Fig. 4e, f). In contrast, there was significant deprotection (i.e., increased deuterium uptake) in both the Fd and LC of var4 compared to Δknob (Fig. 4c, d). Most deprotected peptides are in proximity to the stalk region, with the strongest deprotection in a segment of the LC (residues 88–107) that interacts with the stalk in NC-Cow1 wt (Fig. 4g, h).

**Fig. 5:**
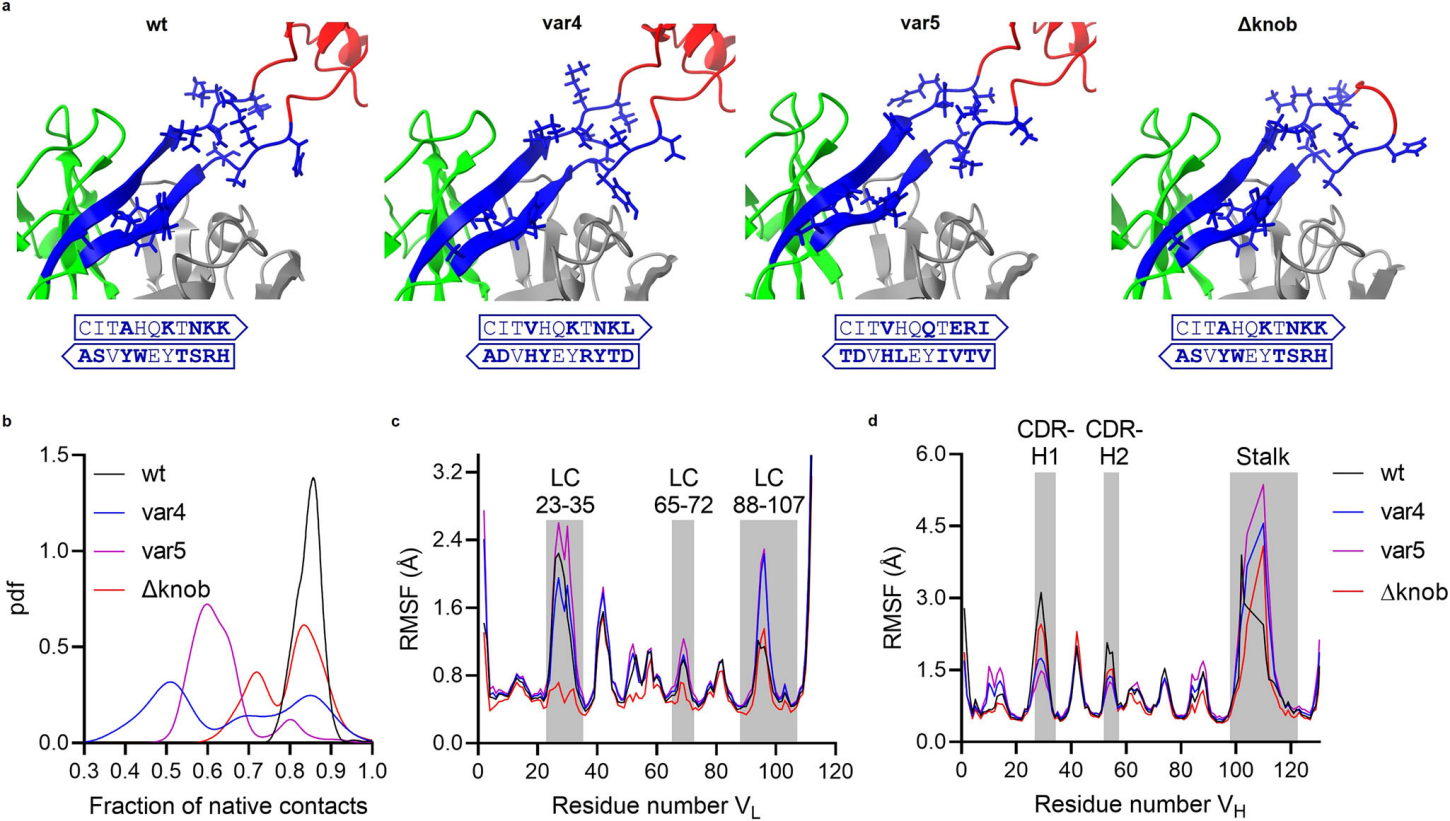
Molecular dynamics simulations of variants with swapped ulCDRs. **a** Simulation snapshots of variants with a zoom-in in the stalk region. The stalk is marked in blue with distinctive residues visualized as single residues and the respective residues marked in bold in ascending and descending stalk representations. The knob region is marked in red, the rest of the V_H_ in green, and the V_L_ in gray. **b** Probability density functions (pdf) for the fraction of native contacts. **c** Root mean square fluctuation (RMSF) per residue of the V_L_. **d** RMSF per residue of the V_H_. The knob region from the CDR-H3 is excluded from the sequence numbering and comparison.

Next, we wondered if molecular dynamics (MD) simulations can confirm the observations from the HDX experiments. To investigate the effect of changes in the stalk region on the dynamics of the antibodies, we performed MD simulations for the heterodimeric variable segment (V_H_–V_L_) of four different constructs (wt, var4, var5, and Δknob). For this, we created mutations of the wt sequence where we exchanged the stalk regions against the stalks of var4 and var5 with a uniform stalk length of 11 ascending stalk residues and 11 descending stalk residues while keeping the scaffold and knob the same (Fig. 5a) (for more information see the “Methods” section and Supplementary Table 3). The knob regions of var4 and var5 were not included as there was no crystal structure as a suitable template for modeling their structure, and we wanted to explore the impact of different stalk regions on interactions with the Fab framework. Importantly, we observed that the changes in the stalk of both var4 and var5 resulted in higher flexibility in the interface of the stalk region. This is apparent from the lower fraction of the native contacts between the stalk and surrounding regions kept during the simulations in these two variants compared to the wt (Fig. 5b). Also, from the root mean square fluctuation (RMSF), the ulCDR region and a segment of the LC in proximity (LC 88–107) are more flexible in var4 and var5 than in wt and Δknob (Fig. 5c, d). This segment of the LC is the same region where we observed significant deprotection for var4 compared to ∆knob in HDX (Fig. 4g), thus further confirming that this region plays an important role in fine-tuning interactions between the LC and the stalk base.

Interestingly, the small differences observed by HDX and MD have only a minor impact on the thermal stability of the Fab variants with different ulCDRs on the same scaffold.

## Discussion

The discovery of unique ulCDR structures in bovine antibodies has expanded the knowledge about the evolution and structural diversity of antibody variable regions. While the very diverse knob domains are important for antigen binding, the stalk is more conserved and critical for stability^9^.

Deleting or replacing the stalk with glycine residues has a very detrimental effect on antibody stability^9^. This could be explained via steric clashes and loss of stabilizing interactions between the stalk and surrounding loops^7,27^. There are variations in the natural ulCDR stalks, and we wondered whether these variations affect antibody stability.

**Fig. 6:**
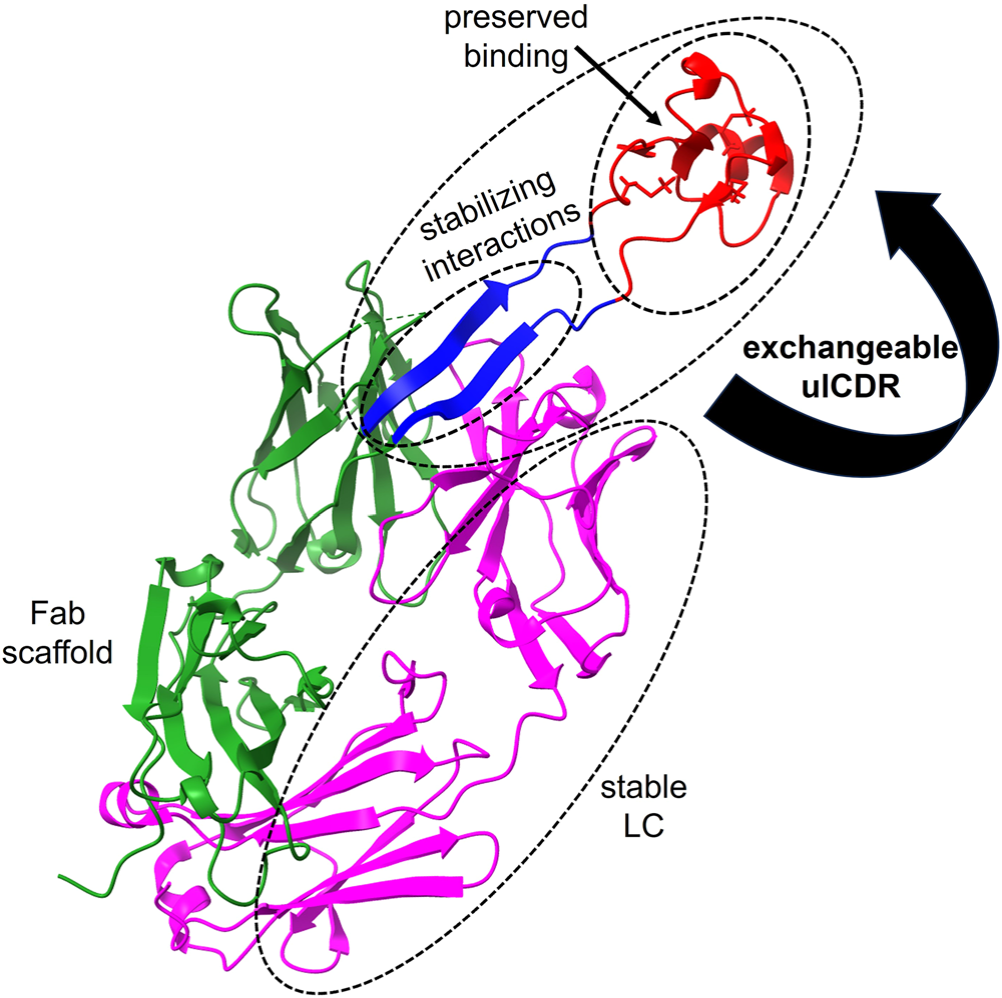
Principles of structural and stability convergence in antibodies with ulCDRs. There is a structural consensus between a Fab scaffold and different ulCDRs. The conserved LC contributes to stability. While the stalk base maintains important stabilizing interactions, the antigen-binding properties of the swapped knob are preserved. Depicted is the crystal structure of NC-Cow1 wt Fab (PDB:6OO0), where the ulCDR is shown as a blue stalk and a red knob, the rest of the Fd region in green, and the LC in pink.

Our work led us to the surprising observation that the overall structure and stability of Fabs with completely different natural ulCDRs were very similar, as illustrated by a narrow range of Fab melting temperatures. This starkly contrasts with the broad distribution of melting temperatures found in conventional antibodies (Supplementary Fig. 17)^28^. Therefore, it is an interesting observation that exchanging an entire ulCDR composed of 50-60 residues has a minimal impact on the secondary structure fingerprints and thermal stability of these antibodies. This suggests that the natural ulCDRs that we tested are compatible with the same Fab scaffold (Fig. 6). In contrast, if the natural stalk residues are replaced with glycine linkers, the thermal stability of the Fab is reduced by ~12 °C^9^. In addition to the similar stability, our comparisons between the ulCDR-swap variants and parent Fabs with ulCDRs show that the high binding affinity of the ulCDR is preserved upon grafting on a different Fab scaffold (Fig. 2, Supplementary Fig. 6). The small reduction in binding affinity of var1 compared to its parent Fab could still suggest a minor impact of the Fab scaffold and the stalk on the positioning of the knob, which would require further investigation.

Despite the overall stability conservation between the different ulCDR-swap mutants, there were minor differences that could be attributed to slightly different amino acid residues in the stalk regions of the different ulCDRs (Fig. 1a). For example, var4 exhibits a less cooperative thermal unfolding than the other variants (Fig. 1f). This observation prompted further structural investigations, revealing that this construct shows deprotection in HDX in the regions that interact with the stalk. In particular, a loop from the V_L_ domain interacting with the stalk showed the highest deprotection. This further supports the notion that the highly conserved LC in these antibodies plays a key role in their stabilization (Fig. 6)^15^. Also, it shows that the small difference in the LC sequences between ulCDR-swap variants and their respective parent Fabs did not negatively impact their stability, thus underscoring the general compatibility of the conserved LC in bovine antibodies with different ulCDRs. Interestingly, other studies have also pointed towards the importance of the LC pairing in bovine antibodies^16,29^. Further experiments with high-resolution methods such as NMR, Cryo-EM, and X-ray crystallography could shed light on interactions between specific residues in the ulCDR and the Fab scaffold.

The special structure of the ulCDR allows it to target a diverse array of antigens, including antiviral, anticancer, and immunomodulatory proteins^18^. This includes the native target of NC-Cow1, the HIV-1 envelope glycoprotein, as well as EGFR and Nkp30. We employed these ulCDRs with different antigen specificities to generate ulCDR-swap mutants against these three antigens and confirmed that target binding could be preserved in these variants with nanomolar affinity. Interestingly, we observed with SEC-MALS that a fraction of var3 and its parent Fab do not form a complex with their antigen EGFR. We collected these unbound Fab fractions and verified with BLI that these fractions do not bind to the antigen. This could be due to a difference in knob disulfide connectivity resulting in different isomers of which only one can bind to its antigen, which, however, does not negatively affect the folding and stability of the whole Fab. The mechanism of knob disulfide formation and its susceptibility to reduction and shuffling will require further investigation. Still, the critical role of properly formed knob disulfides for antigen binding has been demonstrated before^9^.

The unique ulCDR structure has inspired different protein engineering approaches, resulting in novel proteins exhibiting favorable properties such as high-stability and high-affinity binding^18^. Notably, either entire ulCDRs or the respective knob regions can be replaced with different peptides, and ulCDRs or knobs can be grafted onto other scaffolds^9,30–37^. Our study showed that the high sequence diversity of different knobs does not have an impact on overall antibody stability and structure, which supports their potential as functional entities for a wide set of applications in therapeutic protein design. This versatility could also be achieved for humanization purposes. This would require a well-behaved human scaffold with a stalk-like CDR loop^9^.

In conclusion, we demonstrate that natural ulCDRs can replace the ulCDR of one Fab scaffold with minimal impact on the thermal stability and the overall secondary structure of the Fab. In addition, the high-affinity binding to the antigen is preserved. These findings reveal basic structural principles in antibodies with ulCDRs and underscore the potential of these proteins for biomedical applications.

## Materials and methods

### Protein design and expression

The Fab fragments with ulCDRs were obtained by using the V_H_ and V_L_ domains of NC-Cow1 (PDB:6OO0) fused to bovine C_H_1 and C_L_ from BOV-7 (PDB:6E9U), as described before^9^. The design of the ulCDR variants is described in the results section. Plasmids were obtained by commercial gene synthesis from GeneArt (Thermo Fisher)^9^. The same approach was used for obtaining the plasmids for recombinant human EGFR and Nkp30 (extracellular domains with C-terminal 6-His tag) and of the parent Fab fragments of var1^13^, var3, and var4^24^. The plasmids for the HIV-1 Env antigen (BG505 SOSIP.664 gp140-his) and furin were kindly provided by John P. Moore at Cornell University^9^. Larger amounts of the plasmids were obtained by purification with Midiprep and Maxiprep kits (Thermo Fisher) from overnight cultures of transformed XL1-Blue cells, followed by sequencing to confirm the correct inserts. All Fab fragments were produced by transient transfection of Expi293^™^ cells (Thermo Fisher) grown in Expi293^™^ expression medium at 37 °C with 8% CO_2_. The cells were transfected with 0.5 µg plasmid per 1 mL of cell suspension (2:1 LC/Fd plasmid ratio) using the FectoPRO^®^ DNA transfection kit (Polyplus) following the manufacturer’s protocols. For producing the HIV-1 Env antigen, cells were transfected with 1 µg plasmid per 1 mL of cell suspension (4:1 BG505 SOSIP.664/furin plasmid ratio) using the ExpiFectamine^™^ 293 transfection kit according to the manufacturer’s protocols. Recombinant human EGFR and Nkp30 were produced by transient transfection of ExpiCHO™ cells (Thermo Fisher) grown in CHOgro^®^ Expression Medium (Mirus) at 37 °C with 8% CO_2_. The cells were transfected with 1 µg plasmid per 1 mL of cell suspension using the CHOgro^®^ High Yield Expression System (Mirus) following manufacturers’ guidelines. Immediately after the transfection of ExpiCHO™ cells, the temperature was switched to 32 °C until harvest. Cell supernatants from all transfections were collected by centrifugation 4–8 days after transfection. For purification of proteins, an ÄKTA pure (Cytiva) was used at 4–5 °C. The secreted Fab fragments were purified from the cell supernatants by affinity chromatography using a self-packed column with CaptureSelect^™^ LC-lambda (ung) affinity matrix (Thermo Fisher). After a washing step with phosphate-buffer saline (PBS), the bound sample was eluted with 0.1 M glycine pH 3.0 into 1/5 volume of 1 M tris pH 8.5. His-tagged proteins were purified with a HisTrap Excel column (Cytiva). After a washing step with washing buffer (20 mM sodium phosphate, 500 mM NaCl, 20 mM imidazole, pH 7.4), the bound samples were eluted with elution buffer (20 mM sodium phosphate, 500 mM NaCl, 500 mM imidazole, pH 7.4). After affinity chromatography, the samples were further purified by size-exclusion chromatography with a Superose 6 Increase 10/300 GL column (Cytiva) in the case of HIV-1 Env antigen, and a HiPrep Sephacryl S-200 HR column (Cytiva) in the case of all other proteins, using PBS as a running buffer. Finally, the purified proteins were concentrated with Centricon^®^ centrifugal filter devices (Millipore), and the samples were frozen at −80 °C for later use. All samples were stored in PBS pH 7.4.

### Gel electrophoresis

Cell supernatants and purified proteins were analyzed by sodium dodecyl sulfate polyacrylamide gel electrophoresis using SERVAGel^™^ TG PRiME^™^ 4–20% gels and a Dual Color Protein Standard III (SERVA Electrophoresis GmbH). Samples were mixed with 4× Laemmli Sample Buffer (Bio-Rad), and for the reducing gel, DTT (Merck) was added to a concentration of 200 mM. Samples were incubated at 95 °C for 5 min before being transferred into the gel.

### Size-exclusion chromatography coupled to multi-angle light scattering (SEC-MALS)

A Waters 2695 Separation Module HPLC connected to a Waters 2487 Dual Absorbance UV Detector (Waters), a miniDAWN TREOS MALS detector (Wyatt Technology), and an Optilab rEX refractive index detector (Wyatt Technology) was used for most SEC-MALS measurements. For the measurements of var1 or var1PA mixed with HIV Env trimer, var3, var3PA, var4 or var4PA mixed with EGFR, and of var1PA, var3PA, and var4PA alone, an Arc HPLC Quaternary Solvent Manager-R (Waters), an Arc HPLC Sample Manager FTN-R (Waters), a 2489 UV/Vis Detector (Waters), a Fraction Manager (Waters), a miniDAWN TREOS MALS detector and an Optilab refractive index detector (Wyatt Technology) were used. As a running buffer, PBS with 200 ppm sodium azide was used, and the flow rate was 1 mL/min. A Superdex 200 Increase 10/300 GL column (Cytiva) was used for separation. The chromatograms were collected and evaluated using the Astra software v8.1.2 (Wyatt Technology). For molar mass determination, the UV signal with theoretical extinction coefficients was used as a concentration source in the case of the samples containing only Fab fragment, and the RI signal with a constant dn/dc value was used as a concentration source for all other samples.

### Dynamic light scattering (DLS)

A DynaPro plate reader (Wyatt Technology) was used for dynamic light scattering measurements. The measurements were performed in 384 round well low-volume microplates (Aurora Microplates) in triplicates using 30 µl of sample which was sealed with a few µl of silicone oil. Prior to measurement, the plates were centrifuged for 2 min at 2000 rpm. For data collection and processing, the DYNAMICS software version 8.2 (Wyatt Technology) was used. The apparent hydrodynamic radius (*R*_h_) and the onset temperature of aggregation (*T*_agg_) were determined using a protein concentration of 0.5 mg/mL. For isothermal measurements, 10 acquisitions with an acquisition time of 5 s were used for each measurement. For determining *T*_agg_, a temperature ramp of 0.1 °C/min was applied from 25 to 70 °C, and one measurement included 3 acquisitions of 3 s. *T*_agg_ was calculated from the increase in *R*_h_ during heating by the DYNAMICS software.

### Differential scanning fluorimetry (DSF)

Thermal protein unfolding was assessed using a SUPR-DSF (Protein Stable) system that measures intrinsic protein fluorescence intensity. The measurements were performed in 384-well thin-wall Hard-Shell PCR plates (Bio-Rad) in triplicates using 10 µl of sample sealed with Microseal ‘B’ PCR Plate Sealing Film (Bio-Rad). A protein concentration of 0.5 mg/mL was used for measurements. A temperature ramp of 1 °C/min was applied from 10 to 105 °C. Samples were excited at 280 nm, and the barycentric mean within a range of 310 nm to 390 nm was plotted against the temperature. The apparent onset temperature of unfolding *T*_on_ and the apparent melting temperature *T*_m_ were calculated with the SUPR Suite software v3.0 (Protein Stable). For comparing the slopes of the melting curves, a sigmoidal Boltzmann fit was performed using GraphPad Prism version 8.0.1 for Windows, GraphPad Software, Boston, Massachusetts USA, www.graphpad.com.

### Hydrophobic interaction chromatography (HIC)

A Dionex Summit 2 system (Dionex) connected to a UVD170U detector (Dionex) was used. All samples were measured as duplicates using a Proteomix^®^ HIC Butyl-NP5, 5 μm column (Sepax Technologies). Two running buffers were used: Buffer A contained 1.8 M ammonium sulfate and 0.1 M sodium phosphate, pH 5, and Buffer B contained 0.1 M sodium phosphate, pH 5. Samples were prepared by mixing proteins in PBS with Buffer A to result in an ammonium sulfate concentration of 1 M. Each run was done with a flow rate of 0.5 mL/min, consisting of a column equilibration step with 100% Buffer A for 25 min prior to sample injection, a gradient from 0% Buffer B to 100% Buffer B within 40 min, and another 10 min with 100% Buffer B.

### Biolayer interferometry (BLI)

The binding affinity of Fab fragments to antigens was measured using a BLItz system (FortéBio). After an initial baseline of 30 s in PBS, antigen containing a His-tag was captured on an Octet^®^ Anti-Penta-HIS (HIS1K) biosensor (Sartorius) for 300 s at a concentration of 25 ng/µL. Then, after a second baseline of the 30 s, the biosensor sample with immobilized antigen was placed into a solution containing Fab fragment, and the association was recorded for 180 s. After the association step, dissociation was measured for 300 s by placing the sensor into a solution of PBS. Each Fab fragment was measured for antigen binding at 4 different concentrations and normalized to a sample containing only PBS. *K*_D_ values were calculated with fitting functions using the BLItz Pro software (FortéBio).

### Circular dichroism (CD) spectroscopy

Far- and near-UV CD spectra of all protein constructs were acquired on a J-1500 CD spectrometer (JASCO) equipped with a temperature control system coupled with multi-position cells. Near-UV CD samples were acquired at a concentration of 1 mg/ml using a quartz cell with a 1 cm path length. The wavelength was varied from 250 to 350 nm with a 0.1 nm step and an acquisition time of 3 s per point. For each CD spectrum, three scans were averaged and smoothed using the Savitzky–Golay method. Far-UV CD spectra were obtained on 0.02 mg/ml samples using a quartz cell with a 0.1 cm path length. The wavelength was varied from 200 to 280 nm with 0.1 nm step and acquisition time of 3 s per point. For each CD spectrum, three scans were averaged and smoothed using the Savitzky–Golay method^38^. All spectra were processed and analyzed using Spectra Analysis software (JASCO). Secondary structure analysis was carried out with Bestsel software^24^ with a region between 200 and 250 nm included in the fit^39–42^.

### Microfluidic modulation spectroscopy (MMS)

Mid-infrared spectra were recorded with an Aurora (RedShiftBio) using a protein concentration of 1.4–1.6 mg/mL in PBS. Before measurement, the different ulCDR variants were dialyzed against Dulbecco’s phosphate buffered saline (VWR) in a Pierce 96-well Microdialysis plate (ThermoFisher) by following the manufacturer’s protocol. This was to ensure an optimal buffer alignment between the sample and reference buffer. The measurements were performed in 96-well round bottom plates (Corning) sealed with Zone-Free™ Sealing Films (Excel Scientific). Each sample was measured in three replicates, and normalized average absolute absorbance spectra and second derivative spectra were calculated using delta software (RedShiftBio). For the absolute absorbance spectra, a nominal fit displacement factor of 0.6 and a fit range of 1720 cm^−1^ to 1680 cm^−1^ were used. For the second derivative spectra, Savitzky-Golay smoothing was applied using a window of 19 wavenumbers^38^.

### Nuclear magnetic resonance (NMR) spectroscopy

Samples for NMR were prepared by the addition of 5% v/v ^2^H_2_O to 500 μL of a 1 mg/ml solution of proteins and transferred to 5 mm precision NMR tubes (Wilmad). All NMR spectra were acquired at 25 °C on a 600 MHz Avance NEO spectrometer (Bruker) equipped with a 5 mm triple resonance TCI cryoprobe and a temperature control unit. For each sample, 1D 1H spectra were acquired using a standard zggpwg Bruker pulse sequence. The spectra were acquired and processed using Bruker Topspin 4.0.8 (Bruker). Pearson correlation was used to calculate the similarity index between proteins, where regions of the spectra that contain non-protein components were discarded from analysis.

### Hydrogen–deuterium exchange coupled to mass spectrometry (HDX-MS)

Labeling and measurements were performed using an HDX setup from Waters. This includes a PAL RTC Autosampler (LEAP Technologies), a UHPLC with μBinary Pump and Auxiliary Pump (Waters), the HDX Manager of separate column ovens for the pepsin column and analytical column (Waters), and a Synapt XS (Waters). For back exchange, a myoglobin solution of 20 µM in water and a myoglobin solution of 20 µM in D_2_O were prepared. For complete deuteration, the myoglobin in D_2_O was shaken at 35 °C for 1.5 h, and 170 mg NaCl was added to reduce the freezing point. Protein solutions were stored in the quench tray at 1 °C under nitrogen. Three microlitres of protein solution were injected into the labeling vial. Then, 57 μL of labeling buffer (pD 7.4, 5 mM K_2_HPO_4_, 5 mM KH_2_PO_4_, 150 mM KCl in D_2_O) or equilibration buffer (same as labeling buffer, but with H_2_O instead of D_2_O) were added and allowed to react for the set time at 20 °C. Fifty microlitres of the reaction solution were transferred to the quench vial in the quench tray containing 50 μL of quench buffer (pH 2.3, 50 mM K_2_HPO_4_, 50 mM KH_2_PO_4_, 1 M TCEP-HCl, 0.7 M NaOH, 4 M guanidine-HCl in H_2_O) at 1 °C. Fifty microlitres of quenched sample were injected into a BEH pepsin column (Waters) before entering an ACQUITY UPLC BEH C18 VanGuard Precolumn (Waters) coupled to an ACQUITY UPLC BEH C18 column (Waters). Fifty microlitres of the quenched sample were injected into a BEH pepsin column, 5 μm, 30 × 2.1 mm, 300 Å (Waters) and flowed for 1 min with a flow of 75 μL/min, followed by 3 min with a flow of 200 µL/min with 0.2% formic acid in H_2_O at 20 °C before entering an ACQUITY UPLC BEH C18 VanGuard Precolumn, 1.7 μm, 5 × 2.1 mm, 130 Å (Waters) coupled to an ACQUITY UPLC BEH C18 column (Waters) at 1 °C. Thereafter, it started a gradient as shown in Table 2 with the eluent A (H_2_O, adjusted with formic acid to pH 2.5) and eluent B (acetonitrile with 0.3% formic acid) over the trap column to the analytical column ACQUITY UPLC BEH C18, 1.7 μm, 150 × 1 mm, 130 Å (Waters) at 1 °C. The flow rate was 45 μL/min over the entire time. The gradient is a developed and upscaled version of a previously published method^43^. A Waters ESI source was used for ionization with the following settings: capillary voltage of 3.0 kV, source temperature of 90 °C, sampling cone of 50.0 V, source offset of 20.0 V, desolvation temperature of 250 °C, cone gas flow of 100 L/h, desolvation gas flow of 550 L/h, and nebulizer gas pressure of 6 bar. The MS method was UDMSe, with argon as the collision gas and nitrogen as the drift gas. The UDMSe method (Table 3) was developed by taking individual collision energies for the charge states and mass-to-charge ratios based on a previous publication^44^. The wave velocity was ramped linearly from 1500 m/s to 450 m/s with a constant wave height of 40 V, a constant helium gas flow of 180 mL/min, and a constant drift gas flow of 90 mL/min. As LockSpray for recalibration, a solution of 2 ng/μL leucine enkephalin (Waters Corporation) in 50:50 acetonitrile:water with 0.1% formic acid was infused. The labeled protein samples were measured 5 times for each time point at 0.25, 2.5, 13.75, 25, and 250 min, and the protein samples with equilibration buffer with time point 0 min were measured with *n* = 2 for each protein conformation. All measurements with a time point of 0 min were combined as reference. For back exchange, the myoglobin sample in water was measured at *n* = 4 and *t* = 0 s, and the deuterated sample was measured at *n* = 3 and t = 150 s. The evaluation of the analytes was performed using ProteinLynx Global Server and DynamX (Waters). The sequence used for the evaluation was that of NC-Cow1 Fab Δknob for all variants. Based on the peptide fragmentation pattern, a score threshold of 6.60 was chosen for the identified peptides. For myoglobin (back-exchange), the score threshold was 7.70. The intensity threshold was 1000 for the peptides, with a mass error of a maximum of 10 ppm, 0.11 fragments per amino acid, a sum product intensity of 470, and one conducted product^45^. The chromatographic signals between 2.6 min and 14.65 min were evaluated. The cluster data from DynamX was further analyzed using an Excel sheet developed in-house to generate tuptake plots and butterfly plots. This resulted in a sequence coverage of 64.8% with 16 peptides for the Fd and a sequence coverage of 83.3% with 16 peptides for the LC with an average back exchange of 49%.

**Table 2:** Gradient step in the UPLC method from trap column to analytical column

Time (min)	%A	%B
0.00	95.0	5.0
0.50	84.6	15.4
0.90	82.9	17.1
1.36	81.7	18.3
1.82	80.5	19.5
2.27	79.7	20.3
2.73	78.7	21.3
3.18	78.0	22.0
3.65	77.2	22.8
4.09	76.5	23.5
4.55	75.9	24.1
7.27	72.8	27.2
8.18	71.2	28.8
8.64	70.6	29.4
9.09	69.7	30.3
9.54	68.8	31.2
10.00	67.5	32.5
10.50	65.0	35.0
11.00	60.0	40.0
11.50	60.0	40.0
13.00	0.0	100.0
15.00	0.0	100.0

Eluent A was H_2_O, adjusted with formic acid to pH 2.5. Eluent B was acetonitrile with 0.3% formic acid.

**Table 3:** UDMSe method with argon as the collision gas and nitrogen as the drift gas

Bin	Transfer collision energy (V)
1	17.0000
2	17.0000
3	17.0000
4	17.0000
5	17.0000
6	17.0000
7	17.0000
8	17.0000
9	17.0000
10	17.0000
11	17.0000
12	17.0000
13	17.0000
14	17.0000
15	17.0000
16	17.0000
17	17.0000
18	17.0000
19	17.0000
20	17.0000
21	17.1343
22	17.2686
23	17.4029
24	17.5372
25	17.6715
26	17.8058
27	17.9401
28	18.0744
29	18.2087
30	18.3430
31	18.4773
32	18.6116
33	18.7459
34	18.8802
35	19.0145
36	19.1488
37	19.2831
38	19.4174
39	19.5517
40	19.6860
41	19.8203
42	19.9546
43	20.0889
44	20.2232
45	20.3575
46	20.4918
47	20.6261
48	20.7604
49	20.8947
50	21.0290
51	21.1633
52	21.2976
53	21.4319
54	21.5662
55	21.7005
56	21.8348
57	21.9691
58	22.1034
59	22.2377
60	22.3720
61	22.5063
62	22.6406
63	22.7749
64	22.9092
65	23.0435
66	23.1778
67	23.3121
68	23.4464
69	23.5807
70	23.7150
71	23.8493
72	23.9836
73	24.1179
74	24.2522
75	24.3865
76	24.5208
77	24.6551
78	24.7894
79	24.9237
80	25.0580
81	25.1923
82	25.3266
83	25.4609
84	25.5952
85	25.7295
86	25.8638
87	26.0000
88	26.5269
89	27.0538
90	27.5807
91	28.1076
92	28.6345
93	29.1614
94	29.6883
95	30.2152
96	30.7421
97	31.2690
98	31.7959
99	32.3228
100	32.8497
101	33.3766
102	33.9035
103	34.4304
104	34.9573
105	35.4842
106	36.0111
107	36.5380
108	37.0649
109	37.5918
110	38.1187
111	38.6456
112	39.1725
113	39.6994
114	40.2263
115	40.7532
116	41.2801
117	41.8070
118	42.3339
119	42.8608
120	43.3877
121	43.9146
122	44.4415
123	44.9684
124	45.4953
125	46.0222
126	46.5491
127	47.0760
128	47.6029
129	48.1298
130	48.6567
131	49.1836
132	49.7105
133	50.2374
134	50.7643
135	51.2912
136	51.8181
137	52.3450
138	52.8719
139	53.3988
140	53.9257
141	54.4526
142	54.9795
143	55.5064
144	56.0333
145	56.5602
146	57.0871
147	57.6140
148	58.1409
149	58.6678
150	59.1947
151	59.7216
152	60.2485
153	60.7754
154	61.3023
155	61.8292
156	62.3561
157	62.8830
158	63.4099
159	63.9368
160	64.4637
161	64.9906
162	65.5175
163	66.0444
164	66.5713
165	67.0982
166	67.6251
167	68.1520
168	68.6789
169	69.2058
170	69.7327
171	70.2596
172	70.7865
173	71.3134
174	71.8403
175	72.3672
176	72.8941
177	73.4210
178	73.9479
179	74.4748
180	75.0000
181	69.2000
182	63.4000
183	57.6000
184	51.8000
185	46.5000
186	40.2000
187	34.4000
188	28.6000
189	22.8000
190	17.0000
191	17.0000
192	17.0000
193	17.0000
194	17.0000
195	17.0000
196	17.0000
197	17.0000
198	17.0000
199	17.0000
200	17.0000

### Molecular dynamics (MD)

The structure of a bovine Fab NC-Cow1 (PDB:6OO0) was used in this study. We performed molecular dynamics simulations for four different constructs. In the first system (wt), we used the variable segments of the NC-Cow1 (VH and VL). We replaced the knob of the ulCDR with 4 glycines in the second system (Δknob). In the third and fourth systems (var4, var5), we replaced the two stalk regions immediately before and after the knob of the ulCDR with the var4 and var5 versions, respectively. For the replacement, we used the Modeler program^46^. To test the reliability of the replacement modeling, we used two different initial models for var4. They both show similar results in the simulations.

All the constructs were placed in a cubic box simulation using the CHARMM-GUI web server^47^. Sodium and chloride ions were added to make a 150 mM ion concentration. The all-atom CHARMM36m force field was used to study the dynamics of protein, glycan, and ions with the TIP3P explicit model for water molecules^48^. MD trajectories were analyzed using MDAnalysis and VMD^49,50^.

All simulations were performed using GROMACS VERSION 2021.3^51^. The initial setups were minimized for 5000 steps with the steepest descent method and later equilibrated for 500 ps in a canonical (NVT) ensemble and afterward for 7 ns in an isothermal-isobaric (NPT) ensemble under periodic boundary conditions. The positional restraints on initially 4000 kJ mol^−1^ nm^2^ nonhydrogen protein atoms were gradually released during equilibration. Long-range electrostatic interactions were treated with the Particle-mesh Ewald summation^52^ with cubic interpolation and a 0.12-nm grid spacing. During equilibration, the time step was first 1 fs and was then increased to 2 fs during the NPT equilibration. The LINCS algorithm was used to fix all bond lengths^53^. During the equilibration phase, constant temperature and pressure were established with a Berendsen thermostat, combined with a coupling constant of 1.0 ps and an isotropic Berendsen barostat, respectively^54^. The Berendsen thermostat and barostat were replaced by a Nosé–Hoover thermostat and a Parrinello-Rahman barostat during the production runs^55,56^. Analysis was performed on the production simulations. Three simulations were performed for each construct (see Supplementary Table 3 for the description of the simulations).

### Statistics and reproducibility

All experiments are reproducible. The mean values reported are derived from technical replicates. Statistical comparison was used only for the analysis of the HDX-MS data and was performed using a standard and available software, Deuteros 2.0, for significance testing of HDX-MS data.

### Reporting summary

Further information on research design is available in the Nature Portfolio Reporting Summary linked to this article.

## Supplementary information

Supplementary Information

Description of Additional Supplementary Files

Supplementary Data 1

Reporting Summary

## Data Availability

All data supporting the findings of this study are available within the paper and its Supplementary Information. Uncropped images of SDS-PAGE gels are shown in Supplementary Fig. 18. Source data of all graphs and the HDX data are available in the file Supplementary Data 1.
