# Supplementary material for: Stability convergence in natural antibodies with ultra-long hypervariable loops
Source: Commun Biol. 2025 Apr 19;8:635. doi: 10.1038/s42003-025-08036-5 (PMC12009342; doi:10.1038/s42003-025-08036-5)
Supplement: Supplementary file 1 — Supplementary Information [file 42003_2025_8036_MOESM1_ESM.pdf]

Supplementary Information

Stability convergence in natural antibodies with ultra-long hypervariable loops

Marcel Passon[1], Matja Zalar[2], Thomas Nehls[3], Stefaan De Smedt[1], Frederik Lermyte[3], Ahmad Reza Mehdipour[4], Hristo L. Svilenov[1,5,*]

[1]Faculty of Pharmaceutical Sciences, Ghent University, Ottergemsesteenweg 460, 9000, Ghent, Belgium

[2] Faculty of Chemistry and Chemical Engineering, University of Maribor, Maribor, Slovenia

[3] Clemens-Schöpf Institute of Organic Chemistry and Biochemistry, Department of Chemistry, Technical University of Darmstadt, Germany

[4] Center for Molecular Modeling, Ghent University, Ghent, Belgium

[5] Biopharmaceutical Technology, TUM School of Life Sciences, Technical University of Munich, Emil-Erlenmeyer-Forum 5, 85354 Freising, Germany

* To whom correspondence should be addressed:

hristo.svilenov@tum.de

phone: 0049 8161 71 2266

Supplementary Table 1.

ulCDR sequence	Length (aa)	No. Cys	Aggrescan[4] - THSAr	CamSol pH[5] - protein variant score	ProtParam[6] - pI (theoretical)	ProtParam[6] - aliphatic index	ProtParam[6] - GRAVY
NC-Cow1[1] (wt)	62	6	0.049	1.582	6.05	20.48	-1.223
NC-Cow2[1] (var1)	63	6	0.041	0.653	5.79	41.75	-0.949
NC-Cow3[1]	61	6	0.058	0.806	6.04	20.82	-1.213
NC-Cow4/5/6[1]	61	6	0.091	0.577	6.02	31.97	-1.028
NC-Cow7[1]	60	6	0.073	1.271	5.43	42.17	-0.62
NC-Cow8/9[1]	60	6	0.022	1.698	6.06	22.83	-1.073
NC-Cow10[1]	60	6	0.02	1.784	4.47	14.67	-1.227
60B02[2]	62	6	0.052	1.021	5.32	22.1	-0.937
60B07[2]	58	6	0.039	1.142	6.85	30.34	-0.703
60C03[2]	60	6	0.059	0.717	5.48	24.33	-0.795
60C08[2] (var2)	61	8	0.061	0.856	5.79	28.85	-0.728
60C09[2]	56	6	0.041	1.077	5.43	31.43	-0.652
60D01[2]	61	6	0.066	1.261	4.95	24.1	-0.723
60D07[2]	59	4	0	1.292	4.64	24.75	-0.995
60D09[2]	62	6	0.098	0.941	5.32	20.48	-0.939
60E11[2] (var3)	61	6	0.097	1.419	5.45	27.05	-0.957
60F03[2]	62	6	0.052	1.036	4.96	22.1	-0.937
60F06[2]	58	6	0.119	0.579	5.59	35.34	-0.791
60G06[2]	62	6	0.082	0.985	5.32	20.48	-0.982
60G12[2]	62	6	0.123	0.719	5.26	26.77	-0.863
60H05[2] (var4)	62	6	0.059	0.603	7.77	36.13	-0.597
60H10[2]	62	6	0.07	1.337	6.27	20.48	-1.03
63E11[3]	60	4	0.041	1.187	4.96	40.5	-0.733
63A10[3]	53	6	0.113	0.006	6.68	25.66	-0.872
63B08[3]	54	6	0.117	0.914	4.82	45.19	-0.211
63B12[3]	61	8	0.11	0.064	5.57	51.15	-0.272
63C01[3]	55	6	0.057	0.813	5.38	24.73	-0.809
63C05[3]	55	4	0.061	1.572	7.82	42.55	-0.636
63D02[3] (var5)	56	6	0.123	1.013	4.58	62.68	-0.057
63D05[3]	59	6	0.079	0.948	4.96	39.66	-0.454
63D06[3]	57	8	0.074	0.748	6.41	27.37	-0.423
63D10[3] (var6)	59	6	0.075	1.185	7.77	42.88	-0.892
63E04[3]	57	6	0.111	1.059	4.94	42.81	-0.409
63E12[3]	57	6	0.06	1.361	7.73	37.54	-0.909
63F02[3]	55	6	0.044	1.019	4.91	24.91	-0.787
63F07[3]	62	4	0.15	0.573	7.82	44.03	-0.59
63F12[3]	57	6	0.06	1.288	7.75	47.72	-0.821
63H02[3]	62	4	0.105	0.961	7.84	39.35	-0.665
63H12[3]	63	6	0.037	0.923	4.69	12.38	-1.062

Supplementary Table 1. In silico analysis of ulCDR sequences[1–3] with physicochemical descriptors[4–6]. THSAr = Total aggregation hot-spot area per residue. GRAVY = grand average of hydropathicity.

Supplementary Table 2.

Construct	$M_{m,theoretical}$ (kDa)	$M_{m,MALS}$ (kDa)	T_{on} (°C)	T_m (°C)	$R_{h,DLS}$ (nm)	T_{agg} (°C)	K_D (nM)
var1PA	51.8	50.7 ± 0.3	63.0 ± 0.2	70.9 ± 0.1	3.4 ± 0.0	64.2 ± 0.3	81
var3PA	51.2	49.9 ± 0.1	61.9 ± 0.2	74.6 ± 0.2	3.6 ± 0.0	67.0 ± 0.0	15
var4PA	51.3	49.7 ± 0.1	58.5 ± 0.1	71.5 ± 0.1	3.5 ± 0.0	67.1 ± 0.1	41

Supplementary Table 2. Overview of physicochemical descriptors of ulCDR-swap parent Fabs. Mean values with standard deviation are shown. PA = parent antibody.

Supplementary Table 3.

Simulation	Description	Length
WT	The variable segment of bovine Fab NC-Cow1 (PDB:6OO0)	2.4 μs (3 $\times$ 800 ns)
Δknob	Knob is replaced with a short loop of four glycines	1.8 μs (3 $\times$ 600 ns)
var4	Stalk is replaced with var4 sequence	4.15 μs (M1: 3 $\times$ 800 ns & M2: 5 $\times$ 350 ns)
var5	Stalk is replaced with var5 sequence	2.64 μs (3 $\times$ 880 ns)

Supplementary Table 3. Description of the molecular dynamics simulations. M = model.

Supplementary Figure 1.

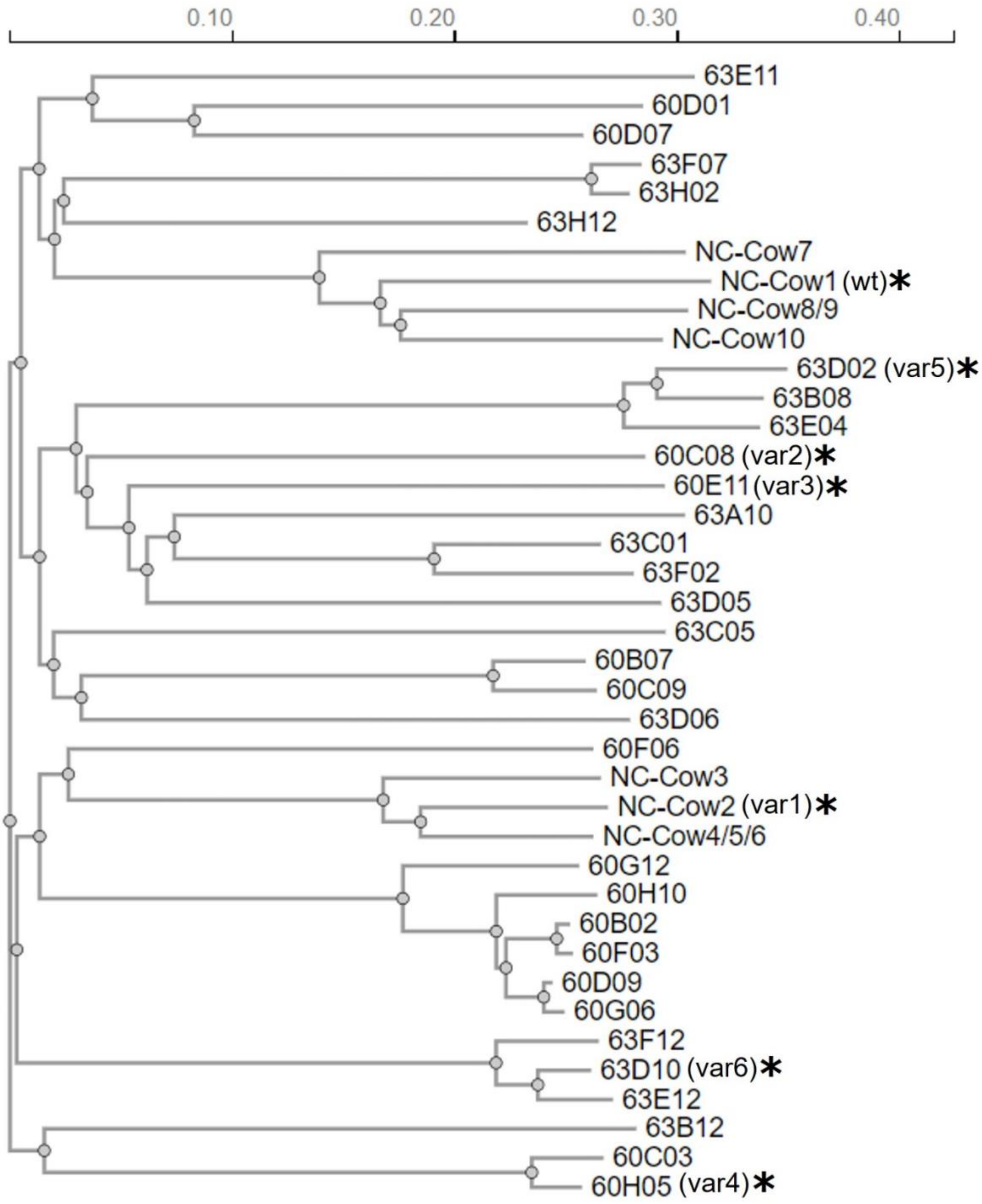

Supplementary Figure 1. Phylogenetic tree of ulCDR sequences. Multiple sequence alignment was done with Clustal Omega.[7] Sequences that were selected for ulCDR-swapping are marked with an asterisk and their respective labeling in this study. The x-axis shows the evolutionary distance between the sequences calculated from the sequence alignment.

Supplementary Figure 2.

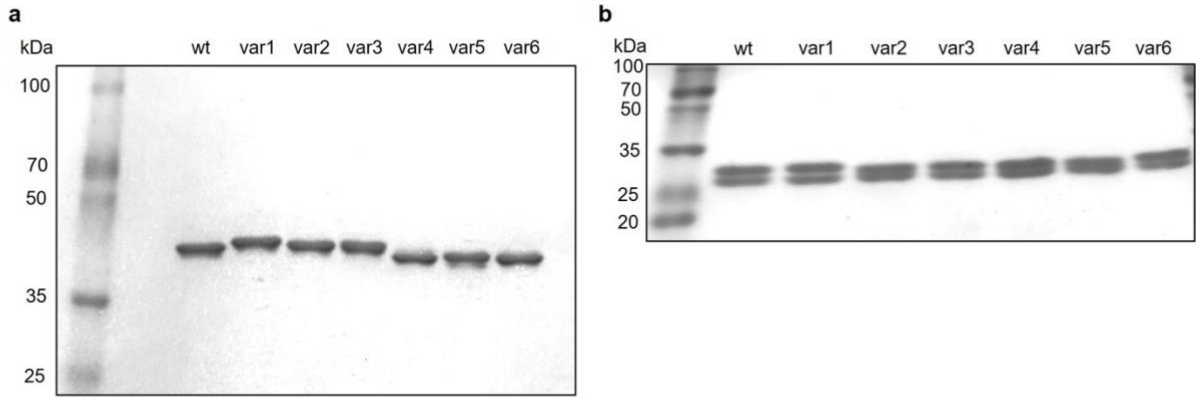

Supplementary Figure 2. SDS-PAGE of purified Fab fragments. a Non-reducing SDS-PAGE. **b** Reducing SDS-PAGE. For reducing SDS-PAGE, a self-prepared polyacrylamide gel with a 4 % stacking gel and a 12 % resolving gel was used.

Supplementary Figure 3.

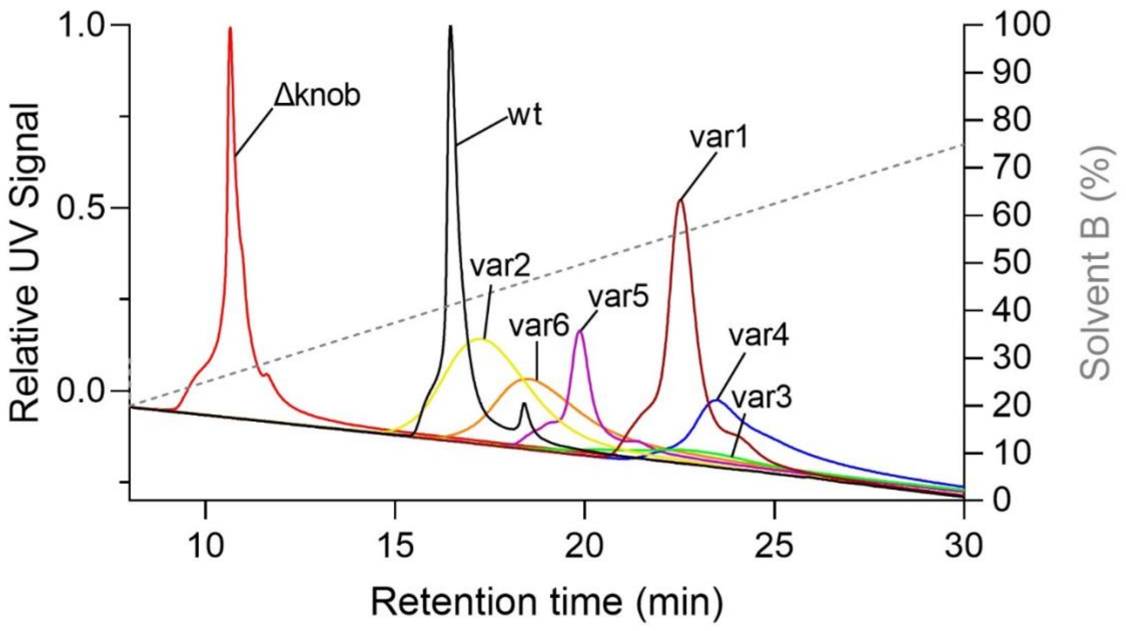

Supplementary Figure 3. HIC chromatogram of purified Fab fragments. Eluting peaks and solvent composition in HIC. Samples were run at a gradient from 0 to 100% Solvent B within 40 minutes.

Supplementary Figure 4.

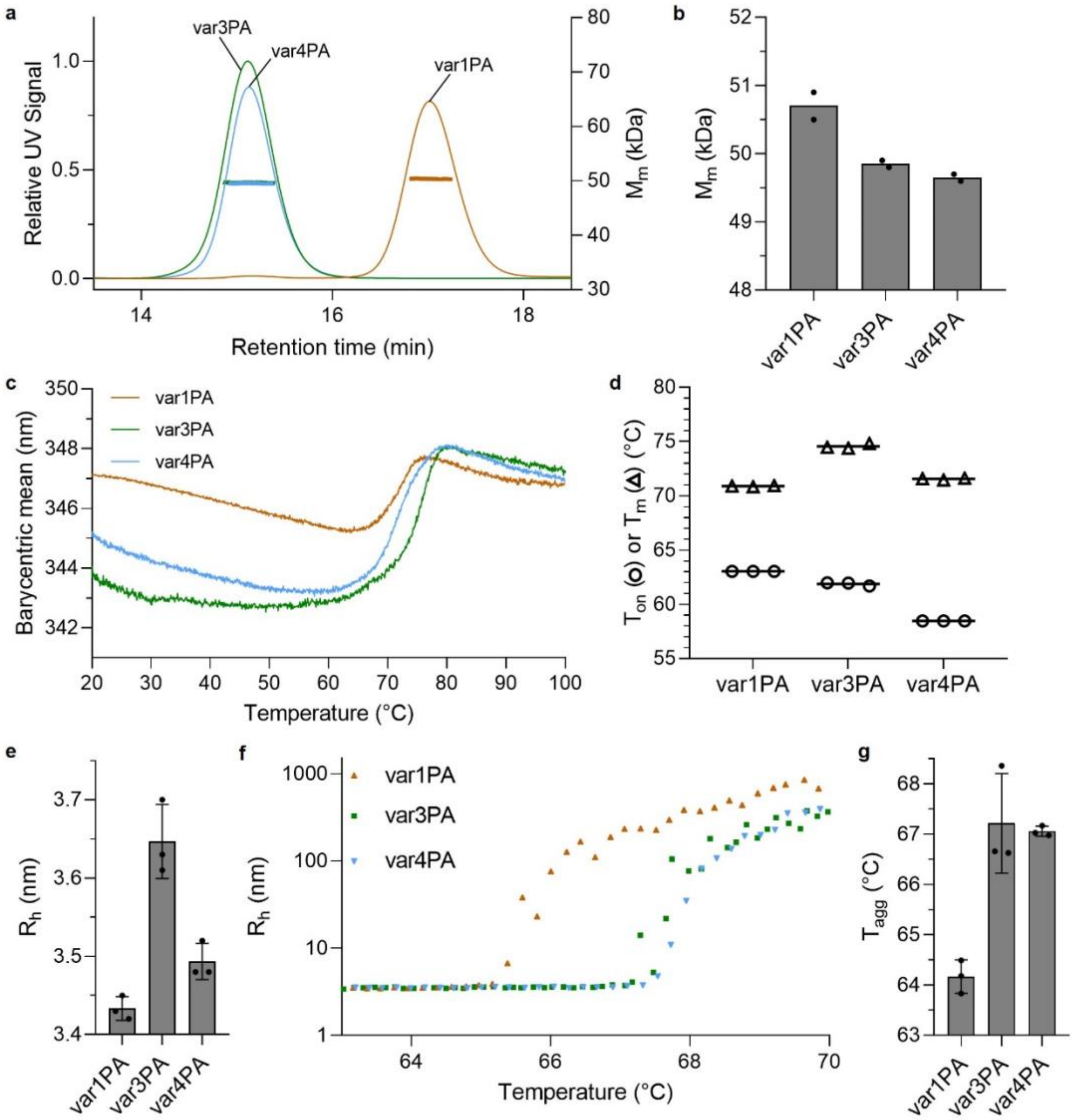

Supplementary Figure 4. Homogeneity and stability of ulCDR-swap parent Fabs. a Molecular mass and eluting peaks in SEC-MALS. **b** Bar chart (n = 2) with molecular masses calculated from MALS. **c** Melting curves in SUPR-DSF. **d** Melting temperatures T_m and onset temperatures of unfolding T_{on} from SUPR-DSF measurements. Mean values and individual replicates (n = 3) (SD shown in Supplementary Table 2). **e** Bar chart with apparent hydrodynamic radii R_h from DLS. Mean values (n = 3) with SD. **f** Graph depicting apparent hydrodynamic radii R_h of heat-ramped samples. **g** Aggregation onset temperatures T_{agg} obtained from heat-ramped DLS. Mean values (n = 3) with SD.

Supplementary Figure 5.

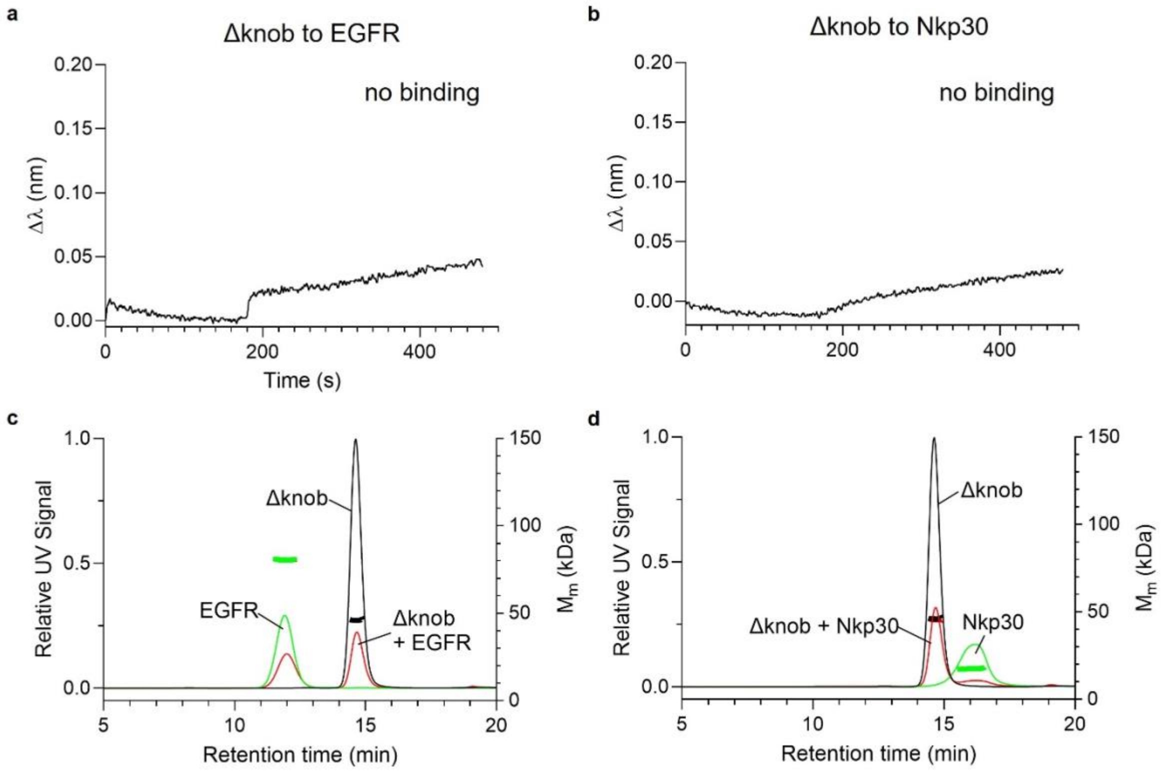

Supplementary Figure 5. No antigen binding of NC-Cow1 Δknob to EGFR and Nkp30. a-b Kinetic measurements of NC-Cow1 Δknob against antigens EGFR and Nkp30. The antigens containing a C-terminal 6His tag were loaded on biosensors and the sensors were dipped into solution containing Fab fragment. No antigen binding was detected at a Fab concentration of 1000 nM. **c-d** SEC-MALS of NC-Cow1 Δknob and antigens EGFR and Nkp30, injected together and alone.

Supplementary Figure 6.

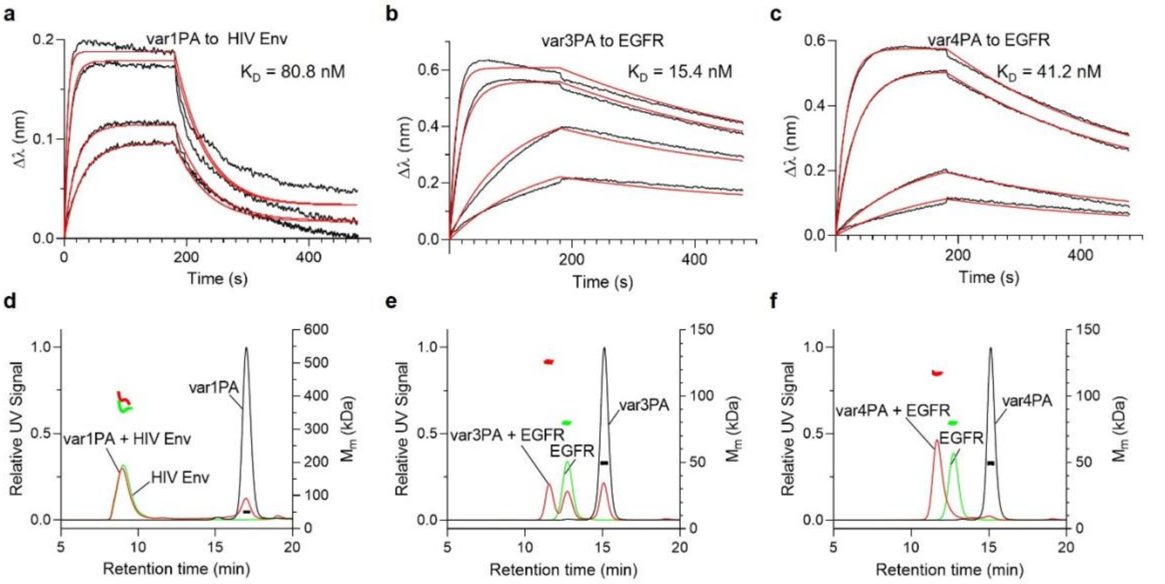

Supplementary Figure 6. Antigen binding of ulCDR-swap parent Fabs. a-c Kinetic measurements of Fab fragments against their respective antigens (HIV Env trimer for var1PA, human EGFR extracellular domain for var3PA and var4PA). The antigens containing a C-terminal 6His tag were loaded on biosensors and the sensors were dipped into solution containing Fab fragment. The antigen binding was measured at different Fab concentrations (100, 200, 500, 1000 nM for var1PA. 50, 100, 500, 1000 nM for var3PA and var4PA.) and fitting functions (in red color) were calculated with the BLItz software. **d-f** SEC-MALS of Fab fragments and their antigens, injected together and alone.

Supplementary Figure 7.

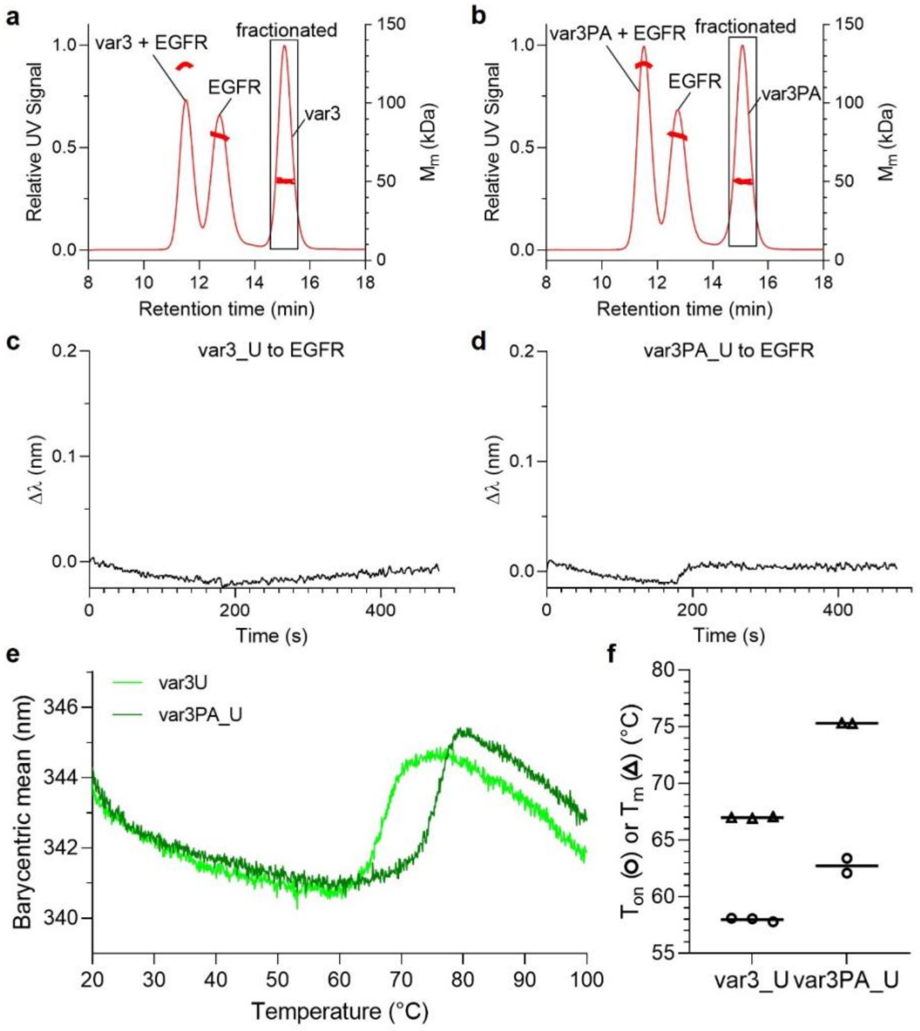

Supplementary Figure 7. Analysis of unbound fraction of var3 and var3PA from mixture with EGFR. a-b SEC-MALS of Fab fragments var3 and var3PA injected together with EGFR. The fractions of unbound Fab fragment were collected for further analysis. **c-d** Kinetic measurements of unbound fraction of var3 (var3_U) and of var3PA (var3PA_U) against antigen EGFR. The antigen containing a C-terminal 6His tag was loaded on biosensors and the sensors were dipped into a solution containing Fab fragment. No antigen binding was detected at a Fab concentration of 100 nM. **e** Melting curves of var3_U (0.18 mg/mL) and var3PA_U (0.24 mg/mL) in SUPR-DSF. **f** Melting temperatures T_m and onset temperatures of unfolding T_{on} obtained from SUPR-DSF measurements. Mean values (n = 3 for var3_U and n = 2 for var3PA_U) are shown.

Supplementary Figure 8.

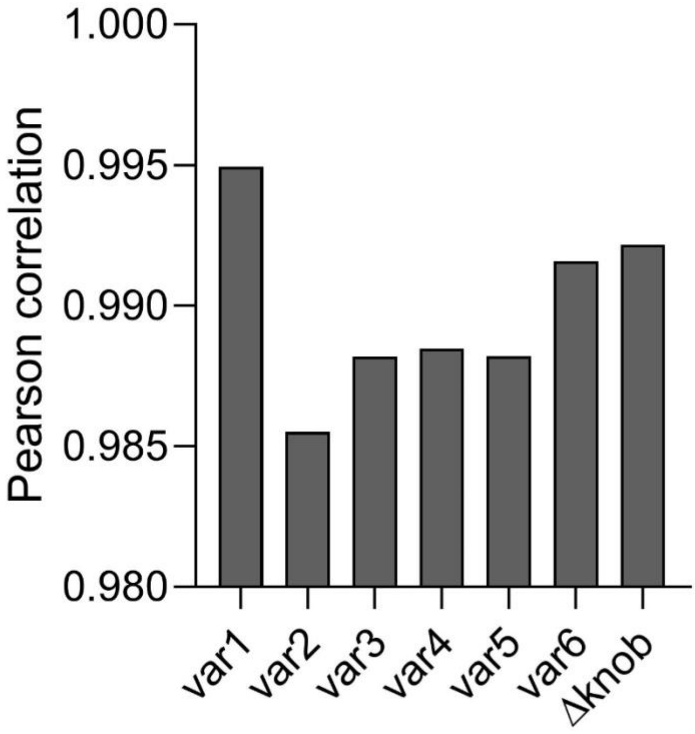

Supplementary Figure 8. Correlation coefficients of 1H NMR spectra of variants relative to NC-Cow1 wt.

Supplementary Figure 9.

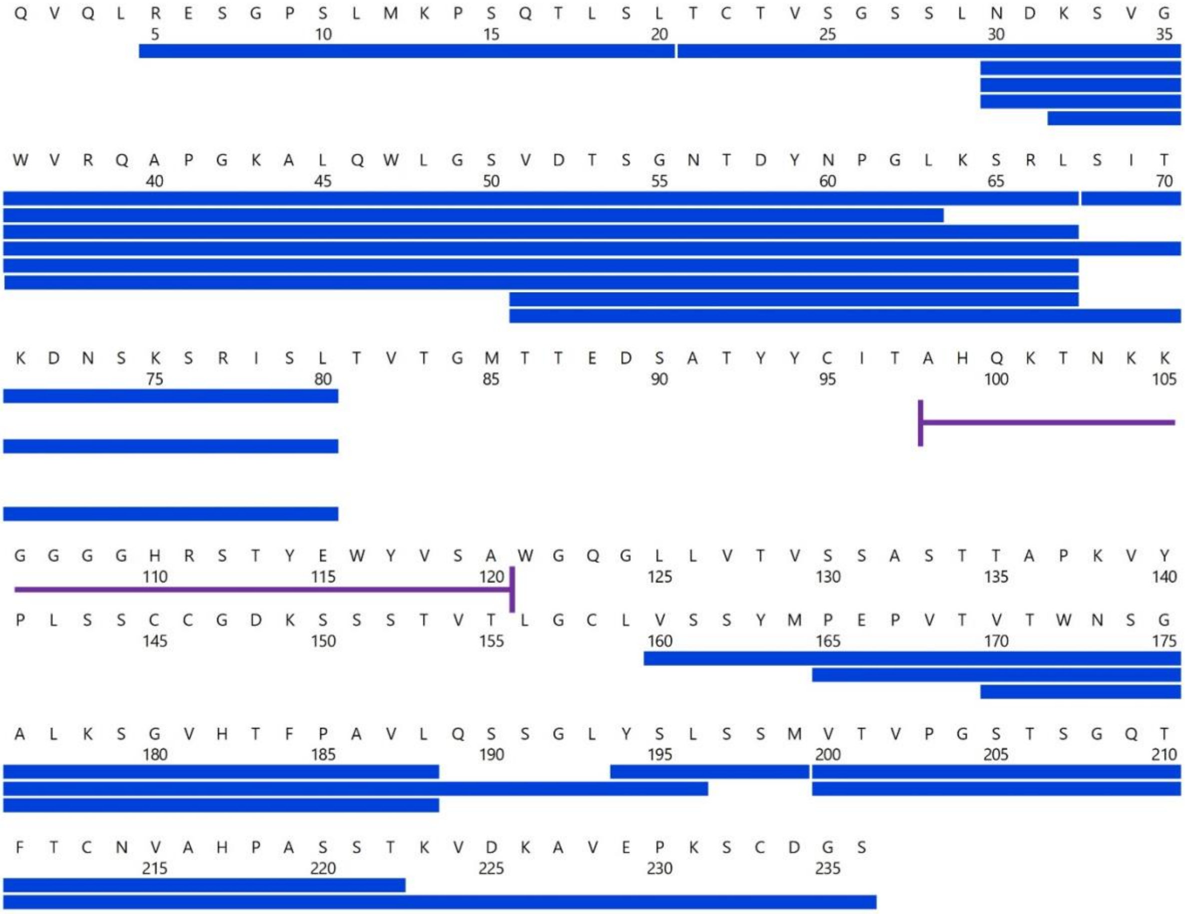

Total: 16 Peptides, 64.8% Coverage, 3.01 Redundancy

Supplementary Figure 9. Sequence coverage of peptides generated in HDX-MS of NC-Cow1 Δknob Fd region. The covered regions are marked with blue bars showing the respective peptides identified. The ulCDR region is marked in purple.

Supplementary Figure 10.

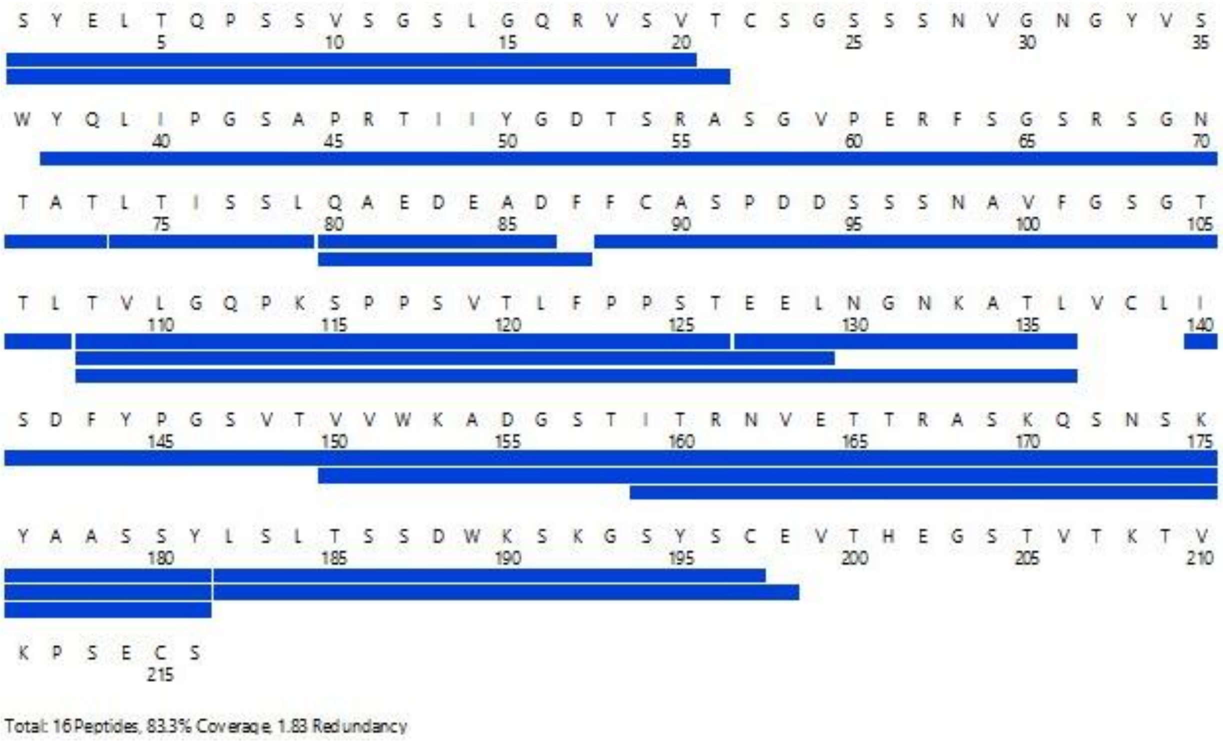

Supplementary Figure 10. Sequence coverage of peptides generated in HDX-MS of NC-Cow1 Δknob LC. The covered regions are marked with blue bars showing the respective peptides identified.

Supplementary Figure 11.

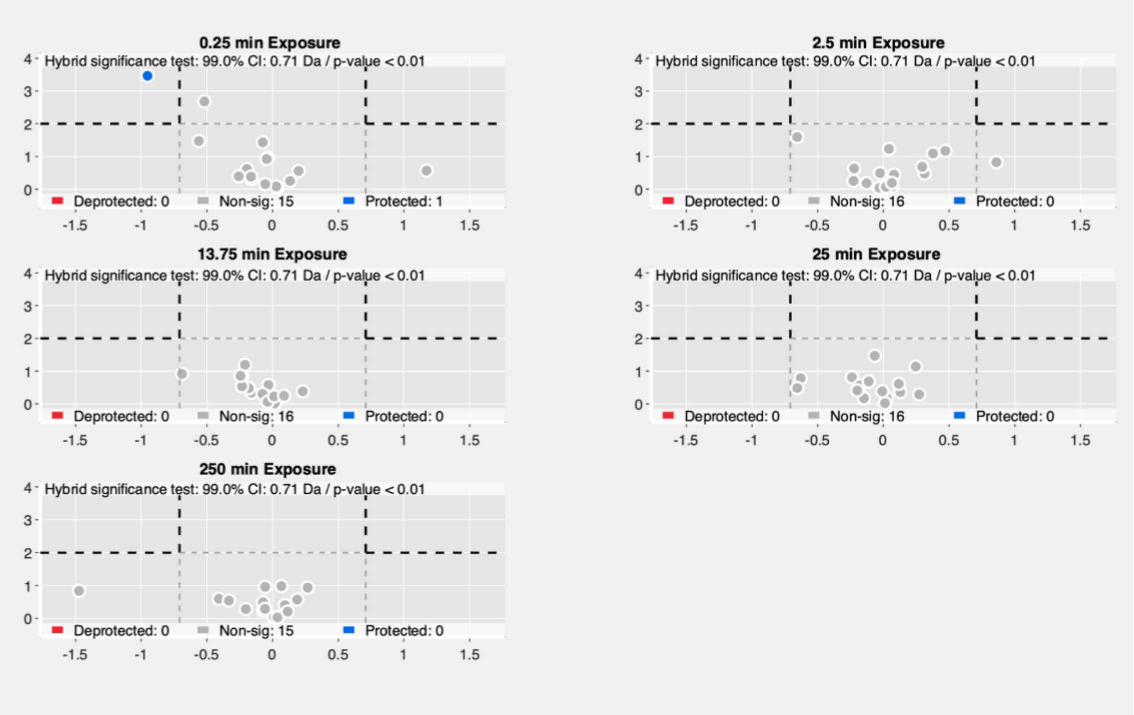

Supplementary Figure 11. Deuterium uptake of NC-Cow1 wt Fd region compared with Δknob for different exposure times. One peptide with significant protection compared to Δknob is marked in blue.

Supplementary Figure 12.

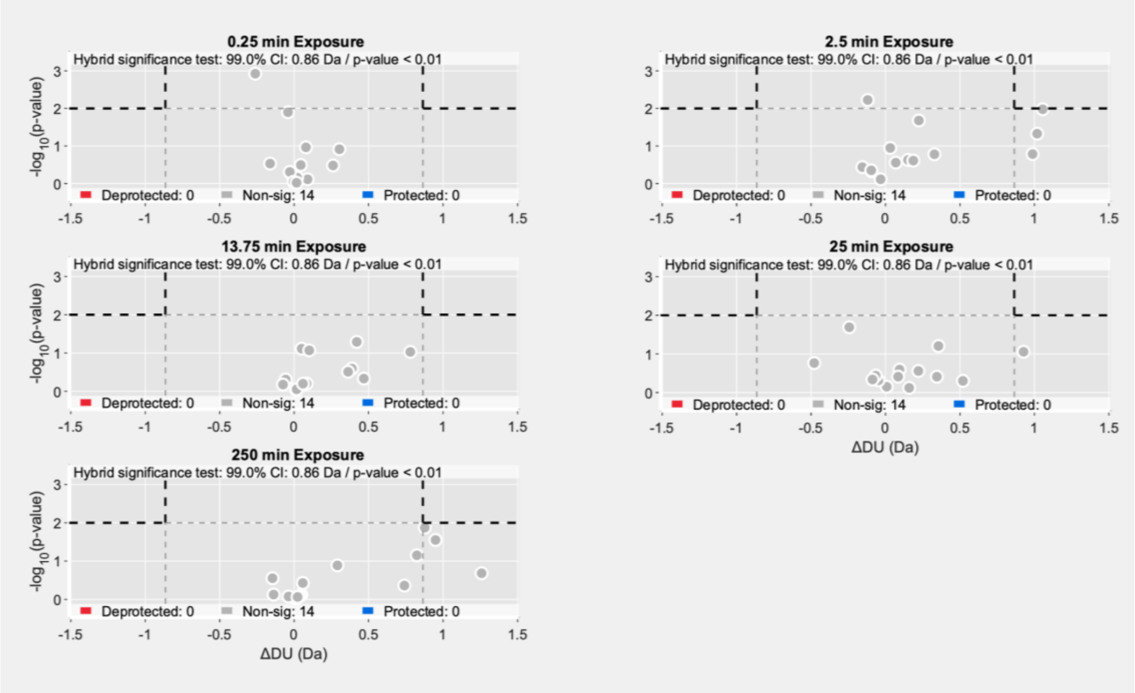

Supplementary Figure 12. Deuterium uptake of NC-Cow1 wt LC compared with Δknob for different exposure times. No peptides with significant protection or deprotection were found.

Supplementary Figure 13.

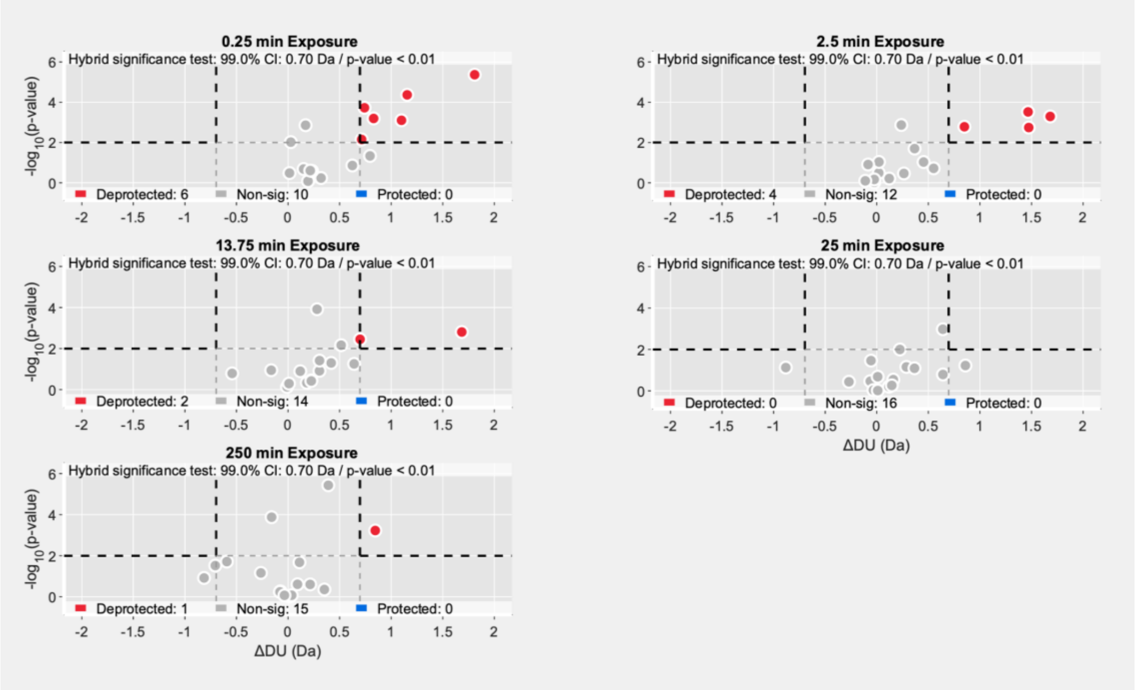

Supplementary Figure 13. Deuterium uptake of NC-Cow1 var4 Fd region compared with Δknob for different exposure times. Peptides with significant deprotection compared to Δknob are in red.

Supplementary Figure 14.

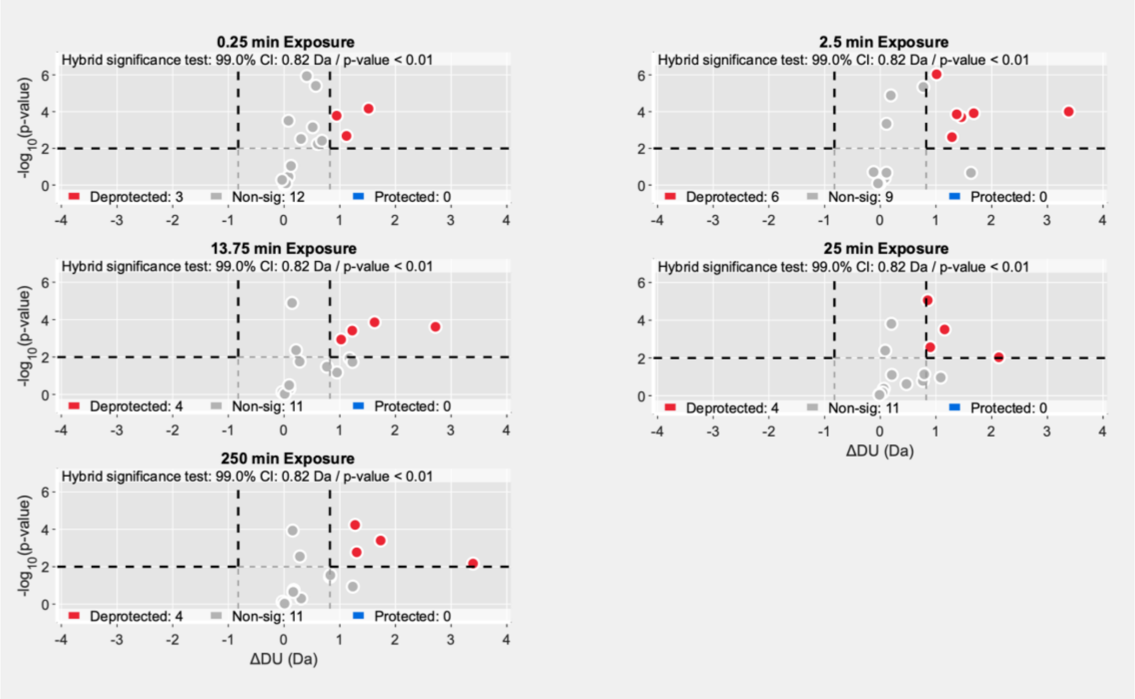

Supplementary Figure 14. Deuterium uptake of NC-Cow1 var4 LC compared with Δknob for different exposure times. Peptides with significant deprotection compared to Δknob are in red.

Supplementary Figure 15.

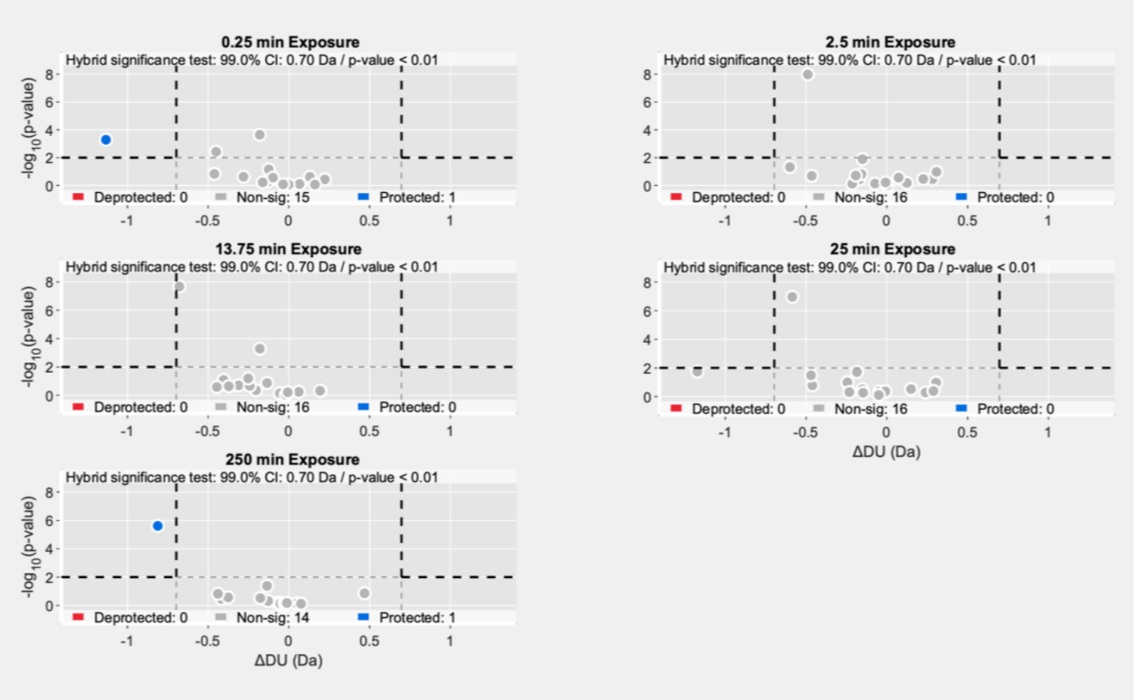

Supplementary Figure 15. Deuterium uptake of NC-Cow1 var5 Fd region compared with Δknob for different exposure times. Peptides with significant protection compared to Δknob are marked in blue.

Supplementary Figure 16.

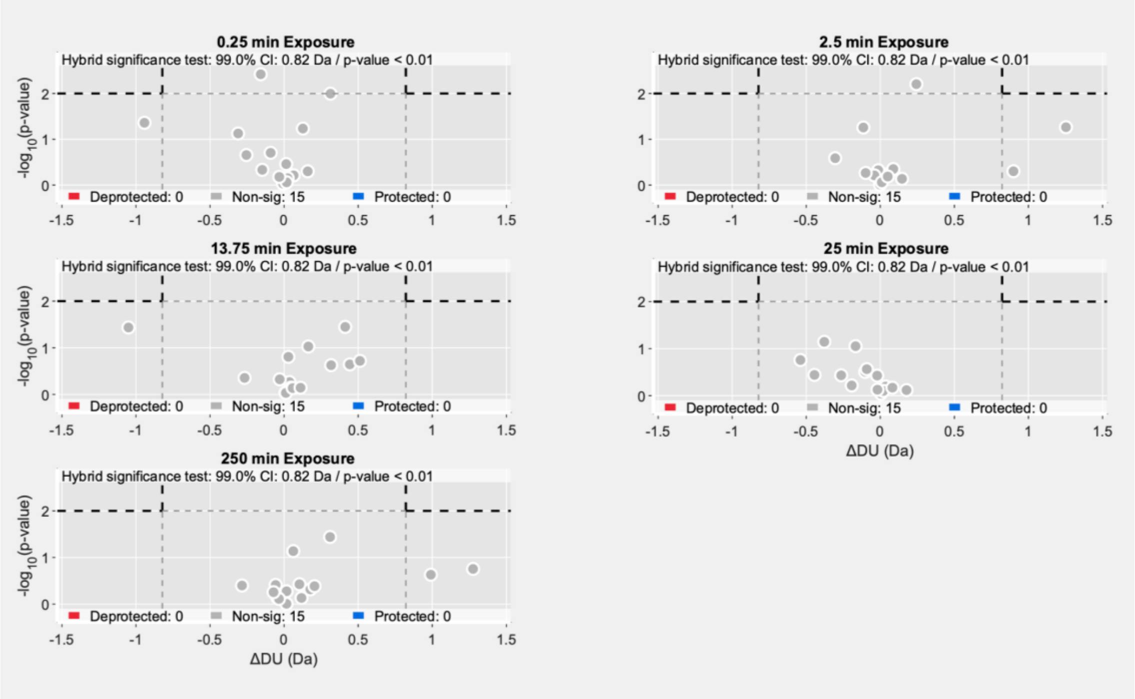

Supplementary Figure 16. Deuterium uptake of NC-Cow1 var5 LC compared with Δknob for different exposure times. No peptides with significant protection or deprotection were found.

Supplementary Figure 17.

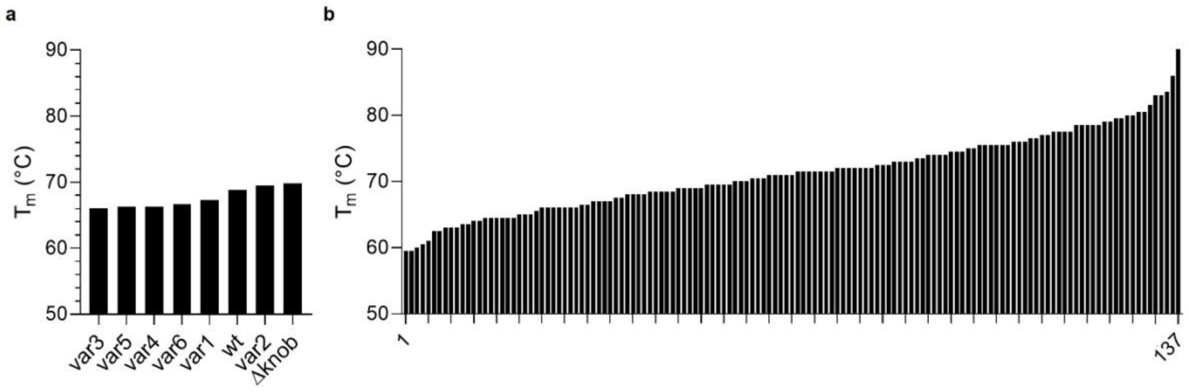

Supplementary Figure 17. Comparison of T_m ranges between **a** ulCDR swap Fab mutants and **b** the Fab in a panel of 137 clinical-stage IgGs.[8]

Supplementary Figure 18.

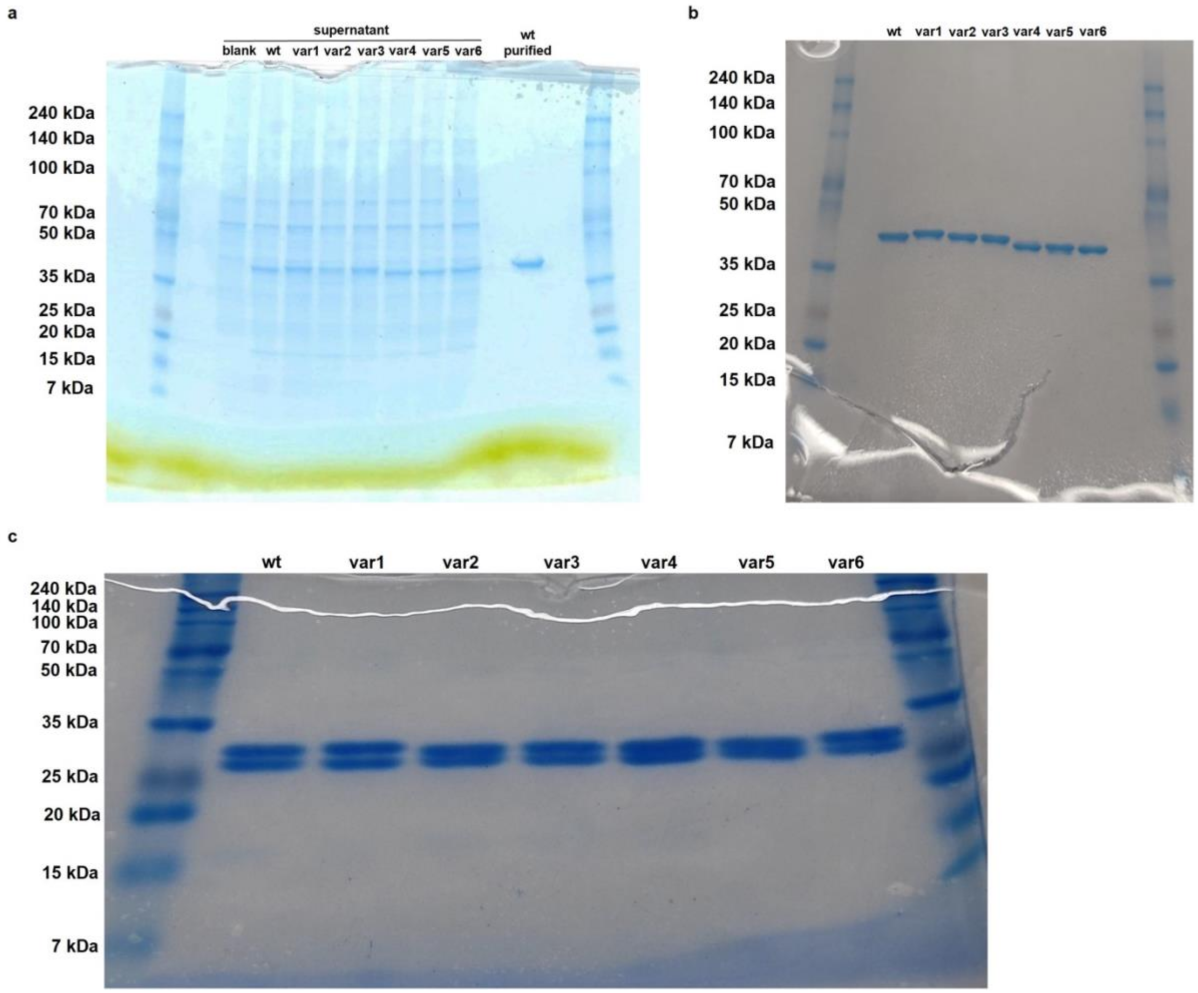

Supplementary Figure 18. Uncropped images of SDS-PAGE gels. a Uncropped image of the gel in Figure 1b. **b** Uncropped image of the gel in Supplementary Figure 2a. **c** Uncropped image of the gel in Supplementary Figure 2b.

Supplementary References

1. Sok, D. *et al.* Rapid elicitation of broadly neutralizing antibodies to HIV by immunization in cows. *Nature* **548**, 108–111 (2017).
2. Pekar, L. *et al.* Milking the Cow: Cattle-Derived Chimeric Ultralong CDR-H3 Antibodies and Their Engineered CDR-H3-Only Knobbody Counterparts Targeting Epidermal Growth Factor Receptor Elicit Potent NK Cell-Mediated Cytotoxicity. *Front. Immunol.* **12**, 742418 (2021).
3. Klewinghaus, D. *et al.* Grabbing the Bull by Both Horns: Bovine Ultralong CDR-H3 Paratopes Enable Engineering of ‘Almost Natural’ Common Light Chain Bispecific Antibodies Suitable For Effector Cell Redirection. *Front. Immunol.* **12**, 801368 (2022).
4. Conchillo-Solé, O. *et al.* AGGRESCAN: A server for the prediction and evaluation of ‘hot spots’ of aggregation in polypeptides. *BMC Bioinformatics* **8**, 65 (2007).
5. Oeller, M. *et al.* Sequence-based prediction of pH-dependent protein solubility using CamSol. *Brief. Bioinform.* **24**, bbad004 (2023).
6. Gasteiger, E. *et al.* Protein Identification and Analysis Tools on the ExPASy Server. in *The Proteomics Protocols Handbook* **112** (ed. Walker, J. M.), 571–607 (2005).
7. Madeira, F. *et al.* The EMBL-EBI Job Dispatcher sequence analysis tools framework in 2024. *Nucleic Acids Res.* **52**, gkae241 (2024).
8. Jain, T. *et al.* Biophysical properties of the clinical-stage antibody landscape. *Proc. Natl Acad. Sci. USA* **114**, 944–949 (2017).
